# An RNA-Binding Complex Involved in Ribosome Biogenesis Contains a Protein with Homology to tRNA CCA-Adding Enzyme

**DOI:** 10.1371/journal.pbio.1001669

**Published:** 2013-10-01

**Authors:** Jinzhong Lin, Jing Lu, Yingang Feng, Mengyi Sun, Keqiong Ye

**Affiliations:** 1 National Institute of Biological Sciences, Beijing, China; 2 Graduate School of Peking Union Medical College and Chinese Academy of Medical Sciences, Beijing, China; 3 Shandong Provincial Key Laboratory of Energy Genetics, Qingdao Institute of BioEnergy and Bioprocess Technology, Chinese Academy of Sciences, Qingdao, Shangdong, China; Brandeis University, United States of America

## Abstract

A multitude of proteins and small nucleolar RNAs transiently associate with eukaryotic ribosomal RNAs to direct their modification and processing and the assembly of ribosomal proteins. Utp22 and Rrp7, two interacting proteins with no recognizable domain, are components of the 90S preribosome or the small subunit processome that conducts early processing of 18S rRNA. Here, we determine the cocrystal structure of Utp22 and Rrp7 complex at 1.97 Å resolution and the NMR structure of a C-terminal fragment of Rrp7, which is not visible in the crystal structure. The structure reveals that Utp22 surprisingly resembles a dimeric class I tRNA CCA-adding enzyme yet with degenerate active sites, raising an interesting evolutionary connection between tRNA and rRNA processing machineries. Rrp7 binds extensively to Utp22 using a deviant RNA recognition motif and an extended linker. Functional sites on the two proteins were identified by structure-based mutagenesis in yeast. We show that Rrp7 contains a flexible RNA-binding C-terminal tail that is essential for association with preribosomes. RNA–protein crosslinking shows that Rrp7 binds at the central domain of 18S rRNA and shares a neighborhood with two processing H/ACA snoRNAs snR30 and snR10. Depletion of snR30 prevents the stable assembly of Rrp7 into preribosomes. Our results provide insight into the evolutionary origin and functional context of Utp22 and Rrp7.

## Abstract 2

The structure of a complex of two ribosome synthesis factors and identification of their ribosomal binding sites provides insights into early stages of ribosome biogenesis.

## Author Summary

Ribosomes are large RNA–protein complexes that manufacture proteins in all living organisms. Synthesis of large and small ribosomal subunits is a fundamental and enormous task that requires activities of approximately 200 assembly factors in eukaryotic cells. These factors transiently associate with the ribosome, forming a series of pre-ribosomal particles. We currently have a poor understanding of the structure and assembly of ribosome precursors. Utp22 and Rrp7 are two interacting proteins present in early precursors of the small ribosomal subunit. In this study, we determined the structure of the Utp22 and Rrp7 complex by X-ray crystallography and NMR and dissected their functional domains by mutagenesis. The structure of Utp22 reveals an unexpected structural similarity to the tRNA CCA-adding enzyme, providing insight into the evolutionary origin of Utp22. Utp22 apparently lacks any enzymatic activity and functions instead as a structural building block. Rrp7 associates extensively with Utp22 and appears to be anchored to pre-ribosomes via a flexible RNA-binding tail. We used RNA–protein crosslinking to identify the binding site and neighboring factor of Rrp7 on pre-ribosomes. Our study provides a detailed insight into the structure of small ribosomal subunit precursors.

## Introduction

Ribosomes, the protein factory, are large RNA–protein complexes composed of small and large subunits (SSUs and LSUs). The core structure and function of ribosomes are universally conserved, but eukaryotic ribosomes are 40% larger than their bacterial counterparts mainly due to the presence of additional ribosomal proteins (r-proteins) and expansion segments in rRNAs [1],[2]. In addition to their greater structural complexity, eukaryotic ribosomes are assembled through a substantially more complex process that involves approximately 200 *trans*-acting protein factors and 75 small nucleolar RNAs (snoRNAs) in the yeast *Saccharomyces cerevisiae*. In contrast, only several tens of ribosome synthesis factors have been found in bacteria [3]. These conserved eukaryotic factors are involved in modification and processing of pre-rRNAs, coordination of rRNA folding, and the assembly of ribosomal proteins and exportation of preribosomal particles from the nucleolus to the cytoplasm (see recent reviews [4]–[7]).

In yeast, ribosome synthesis begins in the nucleolus with the RNA polymerase I–mediated transcription of a 35S pre-rRNA, which encodes 18S, 5.8S, and 25S rRNAs as well as four external and internal transcribed spacers (5′-ETS, 3′-ETS, ITS1, and ITS2). The 35S pre-rRNA associates cotranscriptionally with nearly 50 nonribosomal proteins, U3 snoRNA, and a subset of SSU r-proteins into the enormous 90S preribosome or small subunit processome, which can be visualized as a terminal ball on nascent rRNAs by electron microscopy [4],[8],[9]. Within the 90S preribosome, the 35S pre-rRNA is sequentially cleaved at sites A0, A1, and A2, and these early cleavages can occur during or after transcription [10]. Following these cleavages and a dramatic change in protein composition, a pre-40S particle is released that contains 20S pre-rRNA, which is the 5′-product of A2 cleavage, most of SSU r-proteins, and a handful of nonribosomal factors [11],[12]. The pre-40S particle is exported to the cytoplasm and associates with LSU to complete its maturation [13],[14]. The 3′ product of A2 cleavage, 27SA2 pre-rRNA, is assembled into pre-60S particles and maturates into 5.8S/25S rRNA.

The nucleolus harbors numerous box C/D and box H/ACA snoRNAs that mostly function to guide 2′-O-methylation and pseudouridylation of rRNA. In addition, four snoRNAs in yeast, the U3 and U14 C/D snoRNAs, and the snR30 (U17 in humans) and snR10 H/ACA snoRNAs are required for early processing of 18S rRNA (see review [15]). U3, U14, and snR30 are essential in yeast and thought to be universally conserved in eukaryotes, whereas snR10 is nonessential and appears to be yeast-specific. The former three are known to function by binding to complementary sites on pre-rRNA, but the mode of action remains unknown for snR10 [16],[17].

Most ribosome synthesis factors probably have been identified in yeast through genetic study and biochemical purification of various preribosomal particles. Their association with rRNA processing steps and preribosome types has been generally assigned. The current major challenge is to understand the molecular function of individual factors and the structure and assembly of preribosomal particles. A few factors of the 90S preribosome have been shown to form independent complexes, including UTP-A (t-UTP), UTP-B, UTP-C, U3 snoRNP, the Mpp10–Imp4–Imp3 complex, the Bms1–Rcl1 complex, and the Noc4–Nop14 complex [8],[18]–[22]. Several complexes and factors were shown to assemble into the 90S preribosome in a hierarchical order [23],[24]. Recently, the UV crosslinking and analysis of cDNA (CRAC) method was applied to locate precise binding sites of ribosome synthesis factors on pre-rRNAs [25]–[29]. Cryo-electron microscopy structures have been determined for late pre-40S and late pre-60S particles [12],[30],[31]. However, very little is currently known for structure of early-acting SSU synthesis factors and how and where they associate with 90S preribosomes.

Utp22 and Rrp7 are two proteins associated with early 90S preribosomes and form the UTP-C complex together with four subunits of casein kinase 2 [8],[18]. The UTP-C complex is also associated with transcription factor lfh1 to form the CURI complex, which is implicated in coordination of r-protein production with ribosome biogenesis [32]. Like most early-acting SSU synthesis factors, Utp22 and Rrp7 are required for early processing of 18S rRNA and 40S ribosome formation [33]–[35]. Utp22 and Rrp7 do not contain any recognizable domain, rendering their function highly mysterious.

In this study, we determined the cocrystal structure of the large complex of Utp22 (1,237 residues) and Rrp7 (297 residues) and the NMR structure of a C-terminal fragment of Rrp7, which was not visible in the crystal structure. We found unexpected structural homology between Utp22 and class I tRNA CCA-adding enzyme and between Rrp7 and the RNA-recognition motif (RRM). We identified functionally important domains in the two proteins with structure-based mutagenesis analysis in yeast. We further studied how and where the Utp22/Rrp7 complex assembles into the 90S preribosome. We found that the flexible C-terminal tail of Rrp7 is the key RNA-binding domain that anchors the complex into preribosomes. We mapped the in vivo RNA-binding target of Rrp7 using UV crosslinking and found that Rrp7 binds to the central domain of 18S rRNA and shares a neighborhood with the processing H/ACA snoRNAs snR30 and snR10. We demonstrated that snR30 is required for the stable incorporation of Rrp7 into preribosome. Our comprehensive structure-function analysis of Utp22 and Rrp7 provides important insight into their evolutionary origin and functional context in preribosomes.

## Results

### Structural Determination and Overall Structure of the Utp22 and Rrp7 Complex

**Figure 1:**
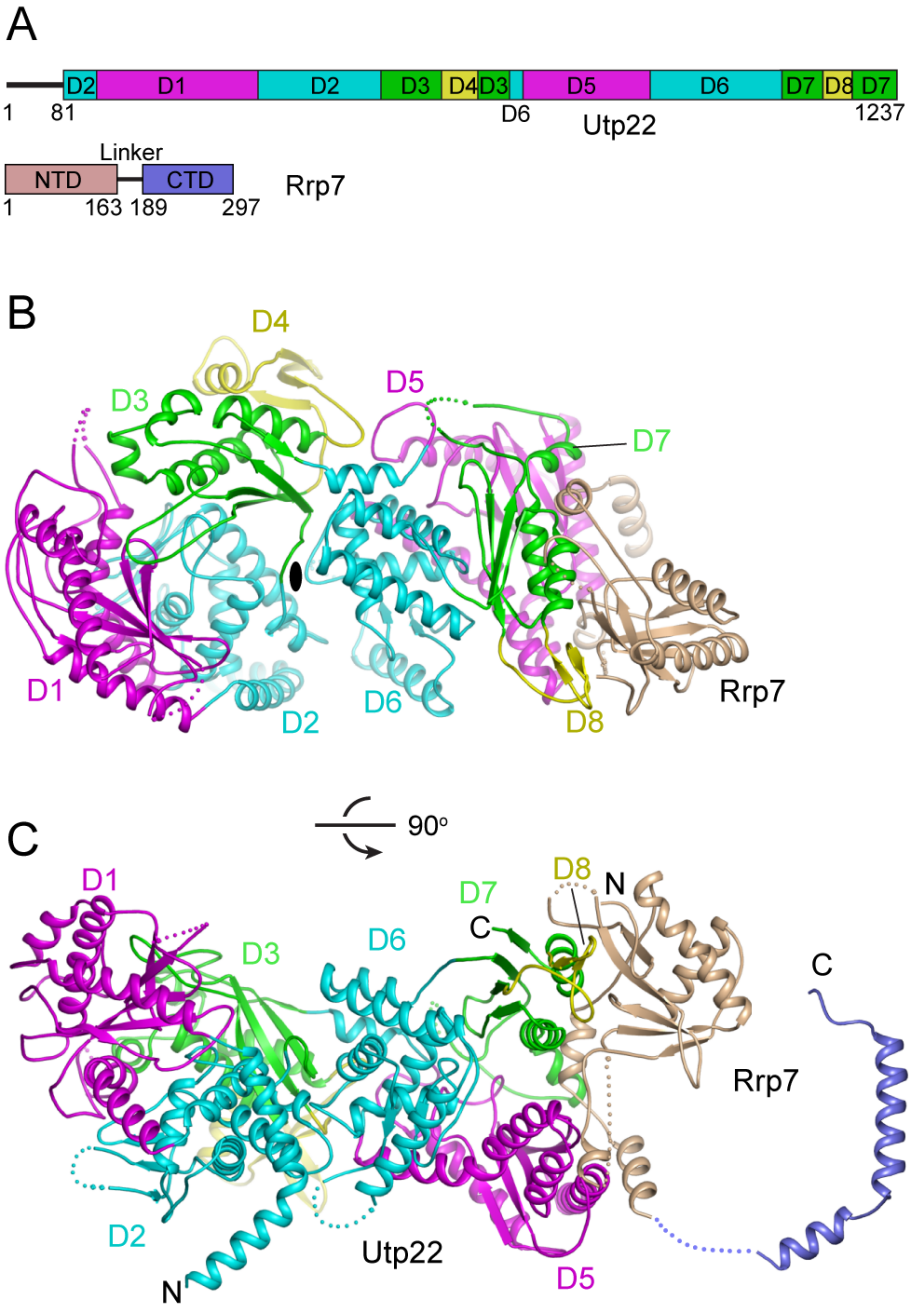
Structure of the Utp22 and Rrp7 complex. (A) Domain diagrams of Utp22 and Rrp7. In Utp22, D1 and D5 are shown in magenta, D2 and D6 in cyan, D3 and D7 in green, and D4 and D8 in yellow. Note that D2, D3, D6, and D7 are each composed of two discontinuous segments. The N- and C-terminal domains (NTD and CTD) of Rrp7 are shown in wheat and blue, respectively. (B) Ribbon representation of the Utp22 and Rrp7 complex structure. Individual domains are colored as described in (A). The same color theme is followed in other figures. Dots represent disordered regions. The view is along the pseudo-dyad axis perpendicular to the paper (shown as ellipse). (C) An orthogonal view. The NMR structure of Rrp7 256–297 is connected by dots to the crystal structure of Rrp7 1–189. The N- and C-termini are labeled.

Both Utp22 and Rrp7 are essential genes in yeast and conserved in eukaryotes (Figure 1A, Texts S1 and S2). However, analysis of their sequences failed to reveal any recognizable domain. We sought to solve the crystal structure of Utp22 and Rrp7 to gain insight into their molecular function. The full-length proteins of yeast Utp22 and Rrp7 were coexpressed using recombinant baculoviruses in insect cells and then copurified and cocrystallized. The structure of the complex was determined by single-wavelength anomalous dispersion phasing based on a Se-labeled crystal and refined to 1.97 Å resolution with an *R*_work_/*R*_free_ of 0.210/0.239 (Table S1). N-terminal residues 1–80 of Utp22, C-terminal residues 190–297 of Rrp7, and several internal loops of each protein were not visible in the crystal structure, likely due to structural flexibility. SDS-PAGE analysis of dissolved crystals showed that Utp22 was intact and Rrp7 was partially degraded (unpublished data).

Utp22 and Rrp7 form a 1∶1 dimer that adopts a saddle-like structure with approximate dimensions of 137 Å×66 Å×69 Å (Figure 1B,C). The N-terminal half (N-half) and C-terminal half (C-half) of Utp22 are structurally similar to each other and are arranged in tandem along the longest dimension. Rrp7 binds at the C-half of Utp22, forming a raised end.

### Utp22 Is a Structural Homolog of Dimeric Class I tRNA CCA-Adding Enzyme

We searched for structural homolog of Utp22 using the DALI server [36] and surprisingly found that Utp22 shares significantly structural homology with class I tRNA CCA-adding enzymes. CCA-adding enzymes are responsible for the synthesis or repair of the universally conserved CCA sequence at the 3′ end of tRNAs [37]. These enzymes catalyze three different polymerization reactions using a single active site and no nucleic acid template. CCA-adding enzymes are classified into two classes: class I is found in archaea, and class II is distributed in eukaryotes and bacteria. Enzymes of both classes are composed of four domains—namely, the head, neck, body, and tail domains. The head domain is the catalytic domain, which is also conserved in the superfamily of nucleotide polymerases. The neck domain constitutes part of the nucleotide-binding pocket, and the body and tail domains bind the tRNA acceptor stem. The two classes share a similar head domain but differ significantly in the remainder of their structures.

**Figure 2:**
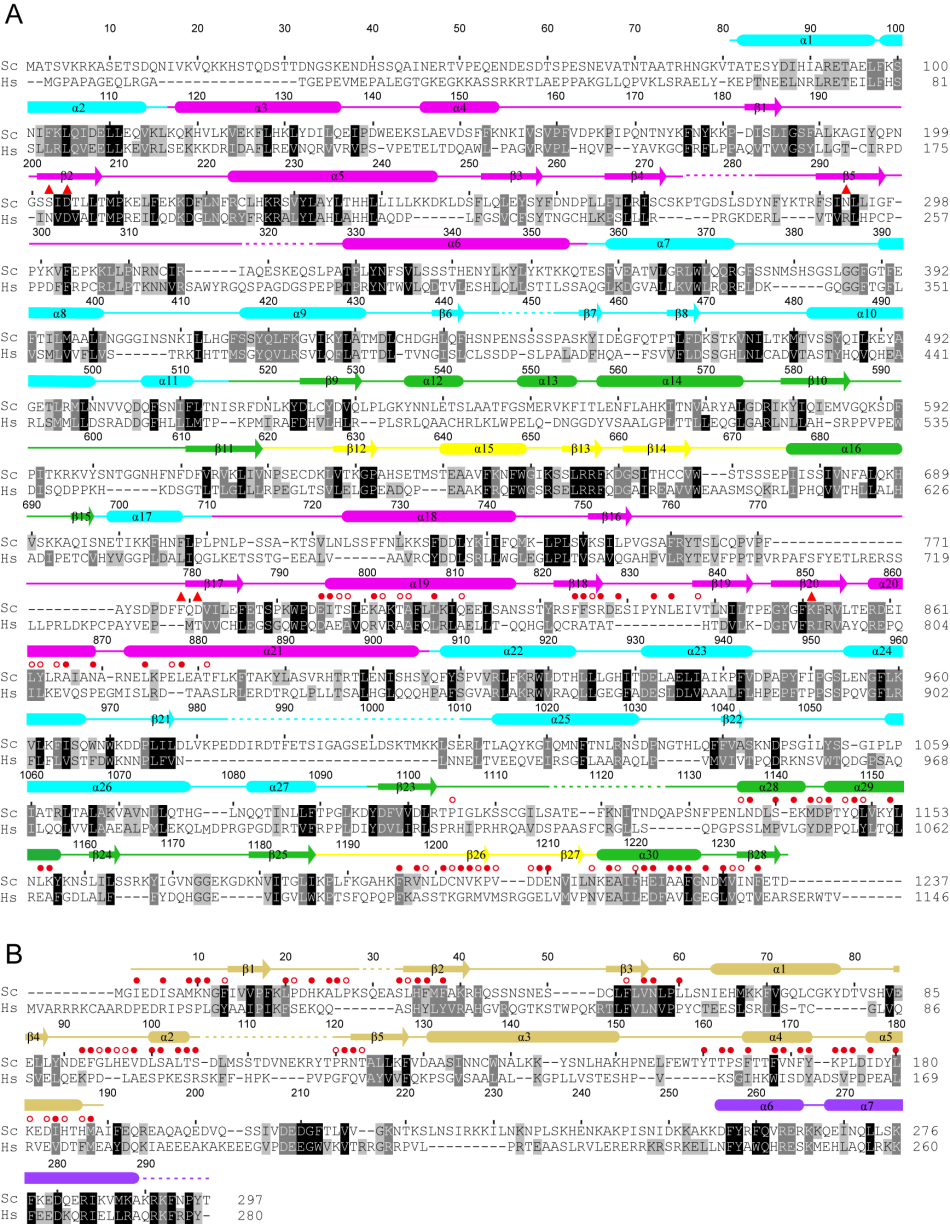
Multiple sequence alignment of Utp22 and Rrp7. Alignment was conducted for 151 Utp22 (A) and 115 Rrp7 sequences (B). Only *Saccharomyces cerevisiae* (Sc) and *Homo sapiens* (Hs) sequences are displayed. Residues that are conserved in 97%, 80%, and 60% of aligned sequences are shaded black, grey, and light grey, respectively. Similarity groups are defined as follows: D and E; K and R; S and T; and F, Y, W, I, L, M, and V. The secondary structures are indicated on the top of alignments and are color-coded by domain as in Figure 1A. Dashed lines denote disordered regions. Residues whose surface areas are buried by 30 Å^2^ and 10 Å^2^ due to the intermolecular association of Utp22 and Rrp7 are marked with solid and empty circles, respectively. Utp22 residues that are equivalent to three catalytic acidic residues in class I CCA-adding enzyme are labeled with solid triangles.

**Figure 3:**
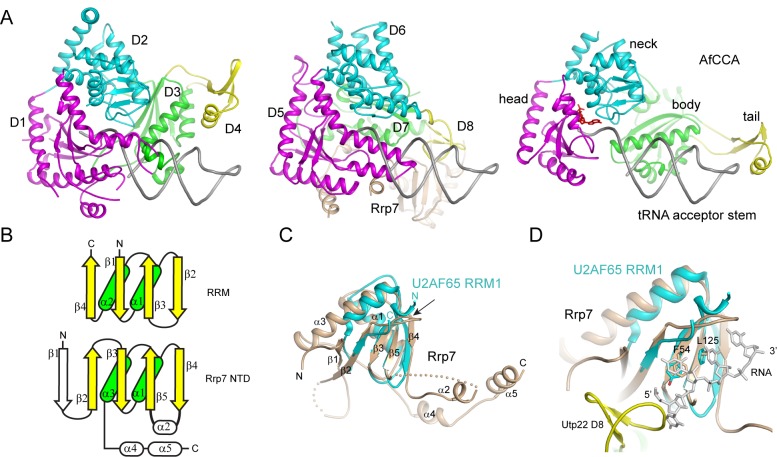
Structural homologs of Utp22 and Rrp7. (A) Structural comparison of Utp22 with AfCCA bound to a tRNA acceptor stem (PDB code 2ZH6). The N-half and C-half structures of Utp22 are aligned to the AfCCA structure based on the head and neck domains and are shown with a modeled tRNA acceptor stem. An ATP molecule bound to the active site of AfCCA is shown as red sticks. (B) Topology diagrams of RRM and Rrp7 NTD, illustrating a cyclic permutation. The shared secondary structural elements are colored in yellow for β-strands and in green for α-helices. (C) The structure of U2AF65 RRM1 (cyan) (PDB code 2G4B) is aligned with the structure of the Rrp7 NTD (wheat). The secondary structures of Rrp7 are labeled. The topology permutation site is marked with an arrow. (D) Putative RNA-binding sites on Rrp7. A polyuridine RNA bound to U2AF65, two RNA-binding residues on U2AF65, and their Rrp7 equivalents are shown as sticks.

The structure of Utp22 can be divided into eight domains (D1 though D8) (Figures 1 and 2A). Both the N-half (D1–D4, residues 81–689) and C-half (D5–D8, residues 699–1237) are structurally similar to class I CCA-adding enzyme in all four individual domains (Figures 3A and S1A–D). We compare the structure of the N-half and C-half with that of *Archaeoglobus fulgidus* CCA-adding enzyme (AfCCA) bound to a tRNA acceptor stem [38]. In the N-half, D1 and D2 combined are superimposable on the head and neck domains of AfCCA. D3 can be aligned with the body domain, but the orientation of D3 with regard to D1–D2 is not conserved. D4 is a small insertion in D3 and shares topology with the tail domain. The four domains in the C-half of Utp22 also bear strong structural similarity with the four domains of AfCCA. Nevertheless, Utp22 and class I CCA-adding enzyme display considerable variations in the length and orientation of secondary structural elements, which precludes detection of their homology based on sequence.

Class I CCA-adding enzymes form a symmetric homodimer. Utp22 is likewise an intramolecular dimer composed of two copies of CCA-adding enzyme modules. The N-half and C-half structures of Utp22 are roughly related by a 180 degree rotation along a pseudo-dyad axis at the interface of D2 and D6 (Figure 1B), and contact each other at D2, D3, and D6 with an extensive interface of 1,621 Å^2^. However, the dimer interface is significantly different between class I CCA-adding enzyme and Utp22 (Figure S1E).

Despite its structural similarity with CCA-adding enzymes, Utp22 is unlikely to possess any polymerase activity. The catalytic domain of CCA-adding enzyme contains three carboxylates (Glu59, Asp61, and Asp110 in AfCCA) that are responsible for binding Mg^2+^ ions and catalyzing the phosphoryl transfer reaction. These catalytic residues are degenerate and nonconserved in D1 and D5 of Utp22 (Figures 2A and S1A–B). The only exception is the Asp204 residue, which is conserved at the corresponding active site of D1. However, Ala substitution of Asp204 did not affect yeast growth (see below), indicating that Asp204 is functionally dispensable. In addition, several structural elements in D1 and D5, including the loop between β5 and α6, the N-terminal part of α6, α20, and the N-terminal part of α21 would occlude the RNA-binding paths in both halves of Utp22 and prevent access of substrate tRNA to the active site. These structural observations indicate that Utp22 is inactive as a CCA-adding enzyme.

### The N-Terminal Domain of Rrp7 Is a Deviant RRM Domain

The structure of Rrp7 is composed of an N-terminal domain (NTD, residues 1–156), a linker region (residues 157–189), and a C-terminal domain (CTD, residues 190–297). The CTD is not visible in the crystal structure. The NTD adopts a two-layered α/β fold, which is similar to the fold of RRM according to DALI search (best *z*-score = 4.8, Figure 3B,C). The structure of Rrp7 NTD can be aligned with that of U2AF65 RRM1 domain with a root mean standard deviation (RMSD) of 1.777 Å over 49 Cα pairs (Figure 3C) [39]. The classic RRM fold has a topology of β1–α1–β2–β3–α2–β4 with juxtaposed N- and C-termini. By contrast, the NTD of Rrp7 displays a cyclic permutation of RRM topology: the strand equivalent to RRM β4 is shuffled to the N-terminus of the strand equivalent to RRM β1. Moreover, Rrp7 has an extra strand β1, which, together with other four β-strands, forms an antiparallel five-stranded β-sheet. Other atypical RRM domains generally have a similar fold as the canonical RRM domain, but differ in RNA-binding mode [40].

The RRM domain is known to recognize single-stranded RNA through its β-sheet surface. Two exposed aromatic (sometimes hydrophobic) residues on strands β1 and β3 are key residues that stack on RNA bases (Figure 3D). The two equivalent residues in Rrp7—that is, Phe54 and Leu125—are conserved (Figure 2B, Text S2) and appear to be accessible for RNA binding. However, replacement of Phe54 to Ala caused no effect on yeast growth (see below), indicating that the putative RNA-binding residue is dispensable. Utp22 associates with the NTD of Rrp7 near the putative RNA-binding site and may interfere with RNA binding. Hence, the RRM-like NTD of Rrp7 is distinct from classic RRM domains in terms of structure and function.

### NMR Structure of a C-Terminal Fragment of Rrp7 Reveals Two Flexible α-Helices

The CTD of Rrp7 is invisible in the crystal structure but is highly conserved and functionally important (see below). We set to determine its solution structure using NMR. A fragment spanning highly conserved residues 256–297 of Rrp7 was expressed in *E. coli* and labeled with ^15^N and ^13^C to facilitate resonance assignment. The structure was determined using 563 NOE-based distance constraints and 68 chemical shift-based backbone dihedral constraints (Figure S2 and Table S2). The structure shows that the C-terminal 40-residue fragment is composed of two α-helices linked by a flexible hinge (Figures 1C and S2B). The two helices are not packed because no long-range NOE was identified between them.

### Dimer Interface Between Utp22 and Rrp7

**Figure 4:**
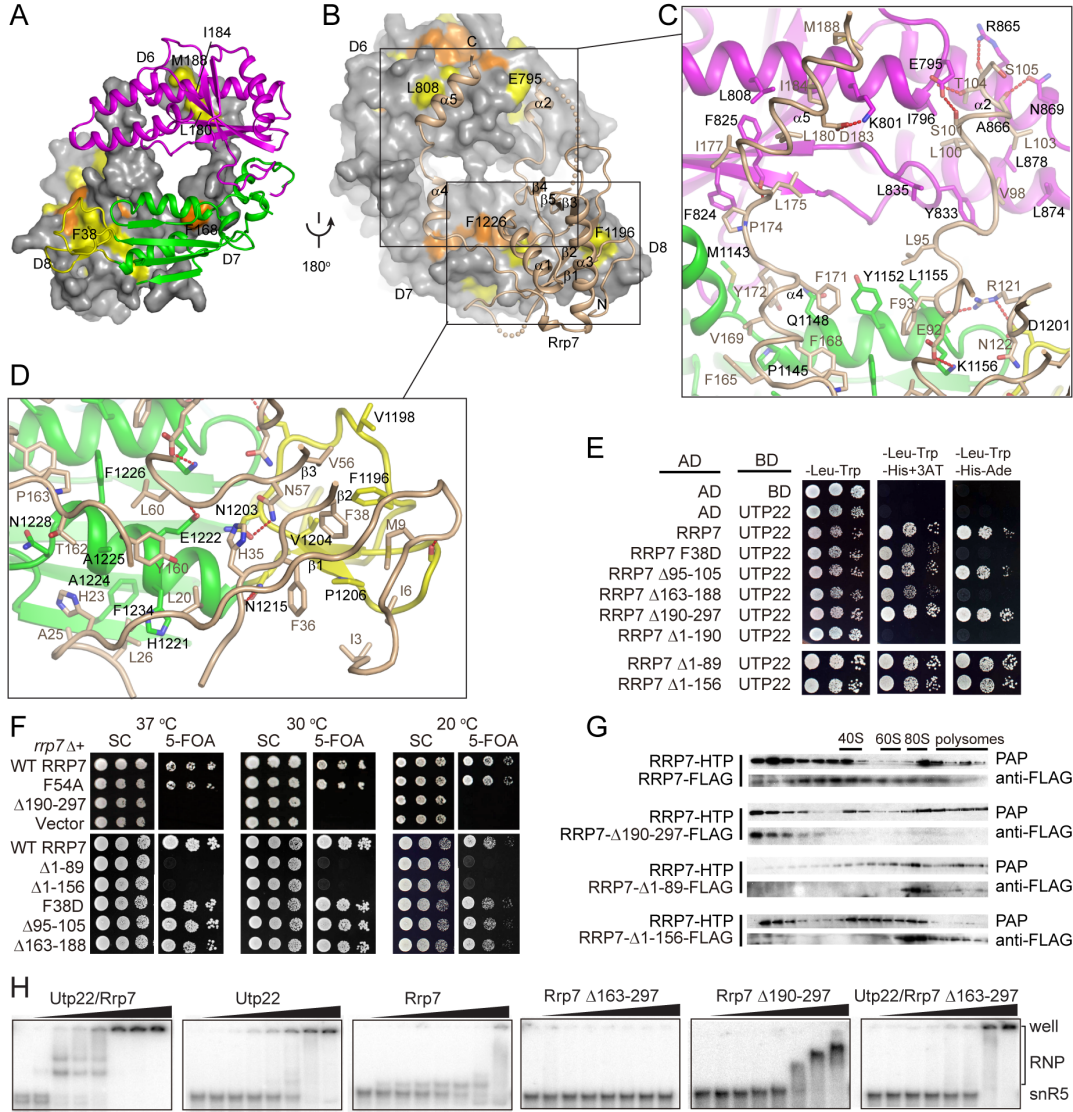
Interactions between Utp22 and Rrp7. (A–B) The binding interface shown in two opposite orientations. Rrp7 is represented as a surface and Utp22 as a ribbon in (A). Utp22 is represented as a surface and Rrp7 as a ribbon in (B). The surfaces are colored in orange for >97% conserved residues and in yellow for 97–80% conserved residues. (C–D) Details of the interaction. Two close-up views corresponding to two boxed areas in (B). Residues involved in intermolecular interactions are shown as sticks. Hydrogen bonds are shown as dashed lines. (E) Two-hybrid interaction between Utp22 and Rrp7. Utp22 was fused to GAL4 DNA-binding domain (BD) as bait, and Rrp7 and its mutants were fused to the GAL4 activation domain (AD) as prey. Ten-fold serial dilutions of AH109 cells containing bait and prey vectors were spotted onto plates containing SC medium lacking Leu and Trp as a growth control, and onto plates containing SC medium lacking Leu, Trp, and His and containing 5 mM 3-amino-1,2,4-triazole (3AT) and onto plates containing SC medium lacking Leu, Trp, His, and Ade to assess protein interaction with increasing stringency. (F) Yeast growth assay of *rrp7* mutants. The *rrp7Δ* strain, complemented by a *URA3 RRP7* plasmid, was transformed with *LEU2* plasmids containing wild-type (WT) *RRP7*, no *RRP7* (Vector), or the indicated *rrp7* mutations. Ten-fold serial dilutions of the transformants were spotted onto plates containing SC medium or SC with 5-fluoroorotic acid (5-FOA) to counterselect for the *URA3 RRP7* plasmid and grown at 37°C, 30°C, or 20°C. (G) Sedimentation behavior of Rrp7 in sucrose gradients. Extracts of *RRP7-HTP* cells overexpressing FLAG-tagged Rrp7 or mutants Δ190–297, Δ1–89, or Δ1–156 from a 2 µ plasmid were fractionated on 7%–50% sucrose gradients and analyzed by SDS-PAGE and Western blotting with peroxidase anti-peroxidase (PAP) and anti-FLAG antibody. (H) EMSA of snR5. 5′-^32^P-labeled snR5 was bound with 0, 100, 150, 200, 300, 400, 600, and 1,000 nM of indicated proteins. Rrp7 189–297 contains a His_6_–SMT3 tag and other proteins have a short His_6_-tag or not.

The NTD and linker region of Rrp7 associate with D6, D7, and D8 of Utp22 through an extensive interface, which buries 3,116 Å^2^ of solvent accessible surface area per subunit (Figure 4A,B). At the center of the interface, one end of the β-sheet of the Rrp7 NTD, which is composed of strands β1, β2, and β3 and surrounding loops, packs against D7 and D8 of Utp22. In addition, two prominent tentacle-like structures project from the NTD to reach the more distant D6 of Utp22. One tentacle comprises a long loop between strands β4 and β5. This loop is disordered in the C-terminal half and its sequence is highly variable among Rrp7 orthologs (Figure 2B and Text S2). The other tentacle is the linker (α4 and α5) that connects the NTD with the flexible C-terminal tail. The intermolecular association is stabilized through a large number of hydrophobic, polar, and electrostatic interactions (Figure 4C,D). Somewhat surprisingly, the dimer interface is only moderately conserved, including the hydrophobic faces on helix α5 and strands β2 and β3 of Rrp7 and a few scattered sites of Utp22 (Figure 4A,B).

### The NTD and CTD of Rrp7 Are Essential for Function

Next, we investigated the contribution of each domain of Rrp7 to Utp22 binding and function. Individual domains of Rrp7 were deleted or mutated and assessed for effect on the interaction with Utp22 using a two-hybrid assay (Figure 4E) and their effect on yeast growth by complementation with the *rrp7Δ* strain (Figure 4F).

Rrp7 and its CTD deletion mutant (Δ190–297) strongly bound Utp22 in two-hybrid assays, whereas the CTD alone (Δ1–190) failed to bind Utp22. This is consistent with the structural observation that the CTD is not involved in the intermolecular interaction. Nevertheless, deletion of the CTD was lethal, indicating that it plays an essential role.

Removal of the β4–β5 loop (Δ95–105)—that is, the Utp22-binding tentacle within the NTD—had no effect on the interaction with Utp22. Deletion of the linker region (Δ163–188)—that is, the other tentacle—reduced the interaction with Utp22 because the two-hybrid reporter strain grew under intermediate stringent but not highly stringent conditions. However, neither tentacle is required for yeast growth (Figure 4F).

Phe38 is located at the hydrophobic interface between the NTD of Rrp7 and Utp22 D5. Substitution of Phe38 with Asp decreased the interaction with Utp22 but did not detectably affect yeast growth, suggesting that the weakened intermolecular association was tolerated. Furthermore, the interaction between Utp22 and Rrp7 was unaffected by removal of either half of or the entire NTD (Δ1–89, Δ1–156). Apparently, the linker region is sufficient for Utp22 association in these cases. The F38D mutation was more disruptive to the interaction with Utp22 than the domain deletion mutations, likely because the negatively charged Asp residue drives the NTD away from the hydrophobic binding face of Utp22 and affects the conformation of the linker region. In contrast with the viable F38D mutation, the NTD deletion mutants cannot support yeast growth, indicating that the NTD has an additional essential function other than Utp22 binding. These results show that Rrp7 and Utp22 are associated with multiple and somehow redundant interfaces and that disruption of a single interface is tolerated in vivo. The redundancy of interface also provides an explanation for its moderate conservation.

### The CTD of Rrp7 Is an RNA-Binding Domain Required for Association with Preribosomes

Given the essential role of the Rrp7 NTD and CTD, we asked whether they function in preribosome association (Figure 4G). We used a yeast strain that expresses His_7_–TEV–ProtA (HTP)-tagged Rrp7 from chromosome as well as FLAG-tagged Rrp7 from plasmid. Sucrose gradient sedimentation analysis shows that wild-type Rrp7 expressed from either chromosome or plasmid was distributed broadly from free protein fractions to large complexes that sediment at positions corresponding to those of 80S to polysomes and should correspond to 90S preribosomes. The CTD deletion mutant of Rrp7 was exclusively present in free protein fractions, indicating that the CTD is necessary for association with preribosomes. Conversely, the deletion of the NTD led to a predominant distribution in 80S-sized and larger particles, suggesting that the NTD is required for the dissociation of Rrp7 from preribosomes.

These mutant Rrp7 proteins were overexpressed under the control of the *GAL1* promoter from a multicopy plasmid. We found that overexpression of the CTD deletion mutant caused slow growth and decreased levels of 40S ribosome, indicating a dominant negative effect (Figure S3). Excessive CTD-lacking Rrp7, which is capable of binding Utp22 but unable to bind preribosomes, would sequester Utp22 in a nonfunctional state. Two NTD truncation mutants (Δ1–89, Δ1–156) displayed no dominant negative effect (Figure S3), likely because they have an incomplete Utp22 binding interface and cannot compete with endogenous Rrp7.

The structural similarities with CCA-adding enzymes and RRM domains suggest that Utp22 and Rrp7 may directly contact RNA in preribosomes. We tested their RNA-binding activity using electrophoretic mobility shift assay (EMSA) with snR5, a yeast H/ACA snoRNA with abundant secondary structures (Figure 4H). Although snR5 is unlikely to be a natural target, such an analysis is useful to identify which protein and domain in the complex are involved in RNA binding. The Utp22 and Rrp7 complex and individual proteins all show at least general RNA-binding activities. The isolated NTD of Rrp7 displayed virtually no RNA binding, but the CTD of Rrp7 still strongly bound RNA. The RNA-binding activity of the Rrp7 CTD may account for its essential role in preribosome association.

### The D2 and D4 Domains of Utp22 Are Functionally Important

**Figure 5:**
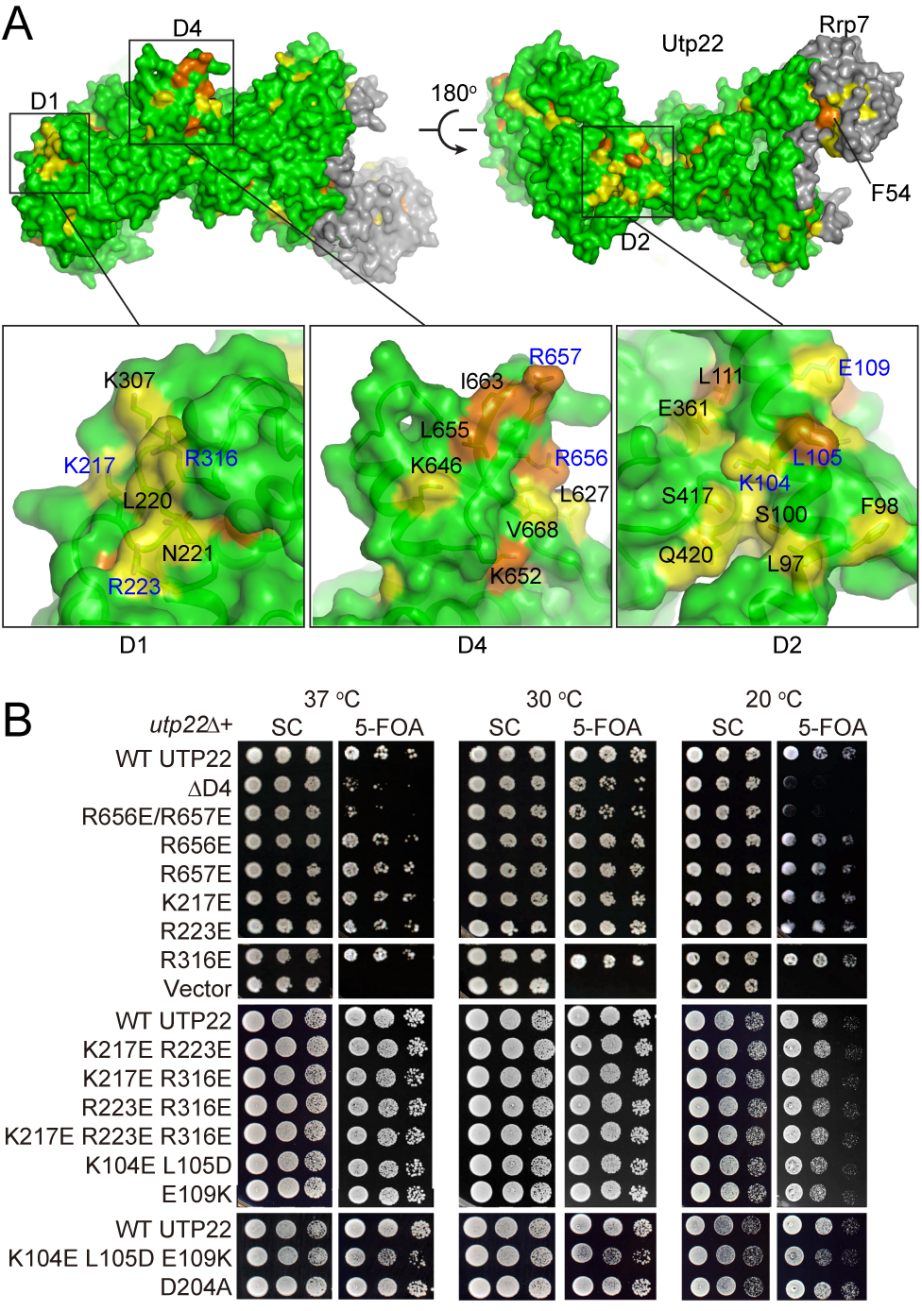
Functional sites of Utp22. (A) Conserved residues on Utp22/Rrp7 surface. Residues that are at least 97% and 80% conserved are colored orange and yellow, respectively. Three boxed regions are zoomed out and shown with semitransparent surface. Residues analyzed by mutagenesis are labeled with blue letters. (B) Yeast growth assay of *utp22* mutants. The *utp22Δ* strain, complemented by a *UTP22 URA3* plasmid, was transformed with *LEU2* plasmids containing wild-type (WT) *UTP22*, no *UTP22* (vector), or the indicated *utp22* mutations. ΔD4 is deletion of residues 630–667. Ten-fold dilutions of the transformants were spotted on plates containing SC medium or SC with 5-FOA to counterselect for the *UTP22 URA3* plasmid and grown at 37°C, 30°C, or 20°C.

To reveal the functional sites of Utp22, we identified the exposed conserved residues on Utp22 structure and assayed their functional importance using mutagenesis and complementation assays with the *utp22Δ* strain. Overall, the exposed surface of Utp22 in the Rrp7 complex is moderately conserved, and there are three conserved patches on D1, D2, and D4 (Figure 5A). The conserved patch on D1 is composed mainly of basic residues. Substitutions of Lys217, Arg223, and Arg316 in this region with negatively charged glutamate, both singly and in combination, had no detectable effect on yeast growth (Figure 5B), indicating that these residues do not play a significant role. The conserved patch on D2 around helix α2 consists of amino acids that have different properties. Incorporation of the L104E/L105D double mutation or the E109K single mutation into helix α2 caused no detectable effect on yeast growth, but the corresponding triple mutation slightly inhibited growth (Figure 5B). This indicates that the conserved patch on D2 is functional.

The small D4 domain protruding from the main body displays the most conserved surface of Utp22. One face of D4 is mixed with highly conserved basic and hydrophobic residues, including Arg656 and Arg657. Although the single mutations of R656E and R657E caused no obvious growth phenotype, the R656E/R657E double mutation inhibited yeast growth at 30°C and inhibited growth more significantly at 37°C and 20°C (Figure 5B). Deletion of the entire D4 domain resulted in a similar degree of growth defect as the double mutation, indicating that these two arginine residues are major functional residues in D4. These results show that D4 is a key functional domain of Utp22.

### Rrp7 Crosslinks to the Central Domain of 18S rRNA and to snR10

The general RNA-binding activity of Utp22 and Rrp7 suggest that they directly bind to the pre-rRNA in preribosomes. We attempted to map their RNA-binding sites using the CRAC crosslinking approach [27]. To this end, the *UTP22* or *RRP7* chromosomal gene was tagged with a C-terminal HTP tag. Following UV-crosslinking in vivo, the HTP-tagged protein was affinity purified via two steps including one conducted under denaturing conditions. The crosslinked RNA was cloned into cDNA and subjected to Solexa sequencing (Table S3). Utp22 crosslinked rather weakly with RNA, and its CRAC result appears to be contaminated by Rrp7-crosslinked RNAs and is therefore not discussed.

**Figure 6:**
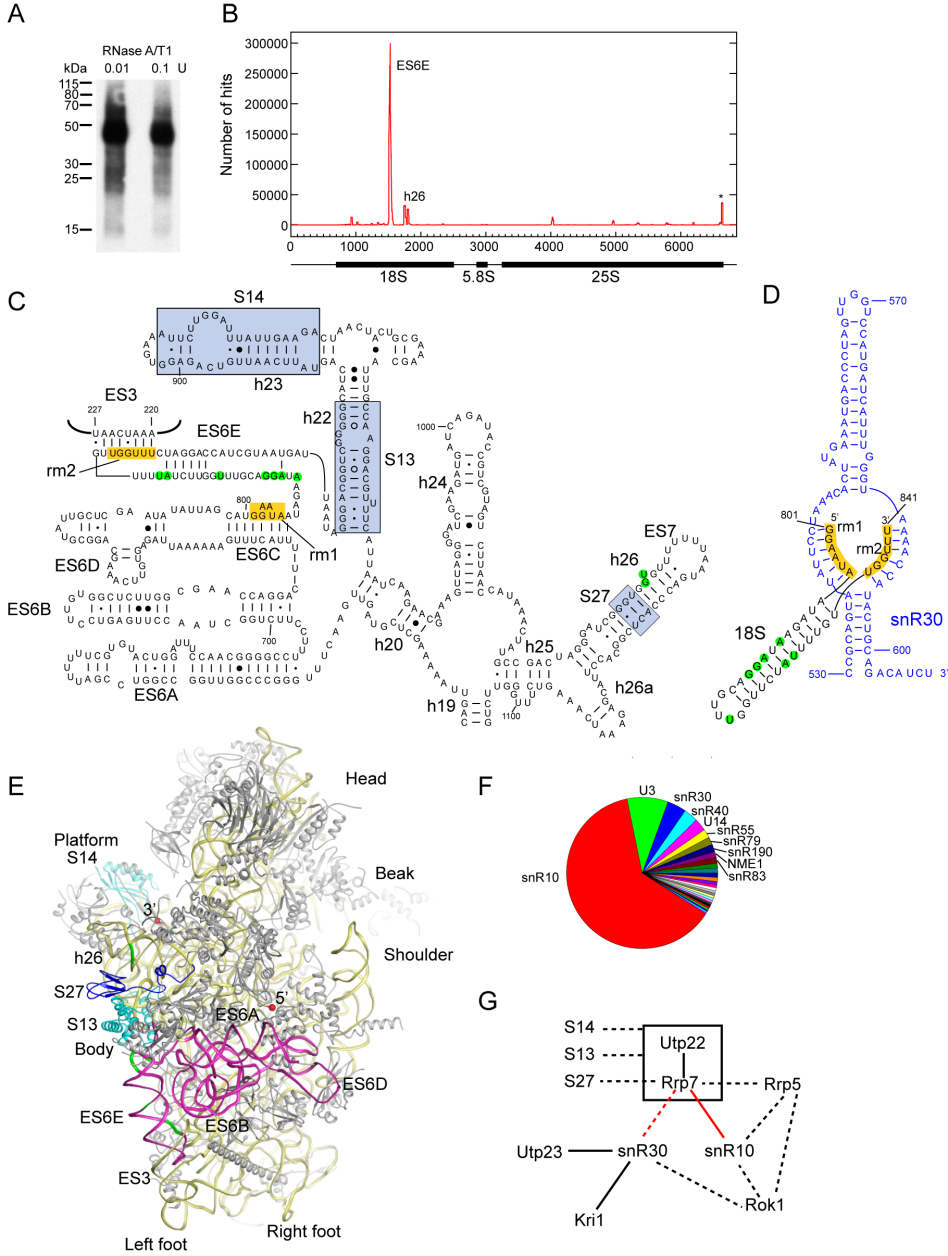
RNA crosslinking sites of Rrp7. (A) Autoradiogram of RNA-crosslinked Rrp7–HTP. Crosslinked RNAs were trimmed with 0.01 or 0.1 U of RNase A/T1 and 5′ ^32^P-labeled. Ni bead eluates were resolved in SDS-PAGE gels and transferred to nitrocellulose membranes, which were then exposed to X-ray films for 2 h. The positions of protein markers are indicated. (B) Pre-rRNA crosslinking sites of Rrp7. Two million reads were aligned to the yeast genome. The number of hits covering each nucleotide of RND37-1 (one of two sequenced rDNA repeats) is plotted. The structure of 35S pre-rRNA is plotted at the bottom. A common contaminant from the 3′-end of 25S is marked with an asterisk. (C) Secondary structure of the central domain of 18S rRNA. Rrp7 crosslinking sites are shaded in green, two snR30-binding sites (rm1 and rm2) are shaded in orange, and binding regions of S13, S14, and S27 are shaded in light blue. Helix numbers and extension segments are labeled. The long-range interaction between ES6E and ES3 is displayed. (D) A model of the interaction between 18S ES6 and snR30. Rrp7 crosslinking sites are marked. (E) Location of Rrp7 crosslinking sites in the yeast 40S ribosome structure (PDB codes 3U5B, 3U5C). 18S RNA is shown in yellow except that ES6 is shown in magenta. Crosslinking sites of Rrp7 are shown in green. S27 is shown in blue, S13 and S14 in cyan, and other ribosomal proteins in grey. Major features of the ribosome and the 5′ and 3′ end of 18S rRNA are labeled. (F) Pie chart of snoRNA reads. The top 10 hits are labeled. (G) An interaction network involving Rrp7, snR30, and snR10. Functional interactions are shown as dashed lines and physical interactions as solid lines. Interactions established in this study are colored in red.

Rrp7 crosslinked efficiently with RNA, as evident by the intense radioactive signal of ^32^P-labeled crosslinked RNA (Figure 6A). Alignment of sequence reads to the reference genome sequence of *S. cerevisiae* revealed that 92.75% of the mapped reads are derived from pre-rRNA (Table S3). A major peak of rRNA reads was mapped to helix E of extension segment 6 (ES6E) of 18S rRNA, and minor peaks were also found in helix h26 (Figure 6B). ES6E and h26 belong to the central domain of 18S rRNA, which constitutes a major part of the platform of ribosome structure and covers the body with ES6 (Figure 6E). One crosslinking peak at 3′-end of 25S rRNA was a frequent contamination [25],[26],[28]. Nucleotide deletions and substitutions in mapped reads are highly indicative of actual crosslinking sites. Such analyses revealed several cross-linking sites on ES6E (nt 812, 814–816, 822, 829–830 based on deletions) and one cross-linking site (nt 1051–1052) on h26 (Figure S4A–C).

Interestingly, the crosslinking region of Rrp7 on ES6E was flanked by two motifs previously found to be targeted by snR30, a conserved H/ACA snoRNA essential for 18S rRNA processing (Figure 6C) [41]–[43]. The two 6-nt motifs, termed rm1 and rm2, are complementary to the bipartite sequences, termed m1 and m2, at the base of an internal loop in the 3′ hairpin of snR30 (Figure 6D) [41].

A small fraction (0.42%) of the mapped reads are derived from snoRNAs (Table S3). Remarkably, 63.6% of snoRNA hits belong to a single snoRNA (Figure 6F), snR10, which is a nonessential H/ACA snoRNA involved in both 18S rRNA processing and pseudouridylation of U2923 in 25S rRNA [16],[17]. The crosslinked RNAs map to nucleotides 190–215 located in the long terminal loop of the snR10 3′ hairpin (Figure S4D,E). The other minor snoRNA hits include processing snoRNAs U3 (8.8%), snR30 (4.1%), U14 (2.7%), and NME1 (1.3%) as well as 24 modification snoRNAs (0.3–3.0%). The significant enrichment of snR10 over other snoRNAs argues that the interaction between snR10 and Rrp7 is real.

### snR30 Is Required for the Stable Association of Rrp7 to Preribosomes

**Figure 7:**
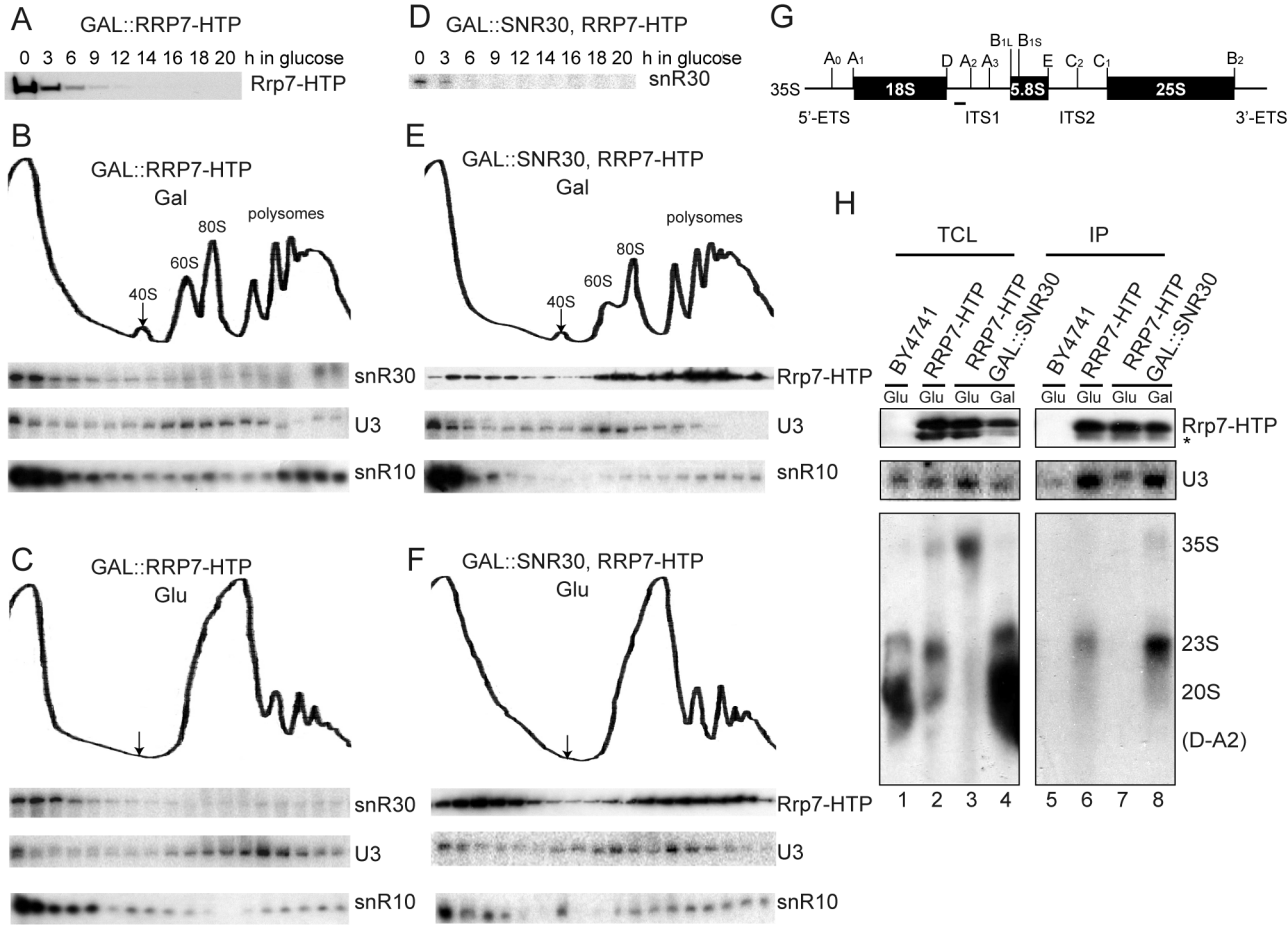
snR30 is required for the stable association of Rrp7 to preribosome. (A) Depletion of Rrp7–HTP in yeast *GAL::RRP7–HTP* after shift to glucose medium. Rrp7–HTP was detected by Western blotting using PAP. Equal amounts of total protein were loaded. (B–C) Sedimentation behavior of snR30 in the presence (B) and absence (C) of Rrp7. Extracts of *GAL::RRP7–HTP* cells grown in galactose (Gal) or glucose (Glu) medium for 16 h were fractionated on 7%–50% sucrose gradients. The distributions of snR30, snR10, and U3 were analyzed by Northern blotting. The polyribosome profiles are displayed. (D) Depletion of snR30 in yeast *GAL::SNR30*/*RRP7–HTP* after shift to glucose medium. snR30 was detected by Northern blotting. Equal amounts of total RNA (1 µg) were loaded. (E–F) Sedimentation behavior of Rrp7 in the presence (E) and absence (F) of snR30. Extracts of *GAL::SNR30/RRP7–HTP* cells grown in galactose or glucose medium for 14 h were fractionated on 7%–50% sucrose gradients. The distributions of Rrp7, snR10, and U3 were analyzed. (G) Schematic structure and cleavage sites of 35S pre-rRNA. (H) Association of Rrp7 with preribosomes. Yeast cells *BY4741*, *RRP7–HTP*, and *GAL::SNR30/RRP7–HTP* were grown in galactose or glucose medium for 14 h. Total cell lysates (TCLs) and immunoprecipitations (IP) of IgG Sepharose were analyzed by Western blotting to detect Rrp7–HTP and by Northern blotting to detect copurifed U3 snoRNA and pre-rRNAs. A probe D-A2 that hybridizes to a region between sites D and A2 was used to detect 35S, 23S, and 20S pre-rRNAs. The minor fast-migrating band of Rrp7–HTP marked by asterisk might be degradation or modification products and its identity was not studied.

The spatial proximity between the binding sites of Rrp7 and snR30 on 18S ES6 raises a question as to whether they are dependent on each other to bind preribosomes. To examine whether association of snR30 with preribosomes depends on Rrp7, the HTP-tagged *RRP7* chromosomal gene was placed under the control of the *GAL1* promoter, which is active in the presence of galactose and repressed in the presence of glucose. The accumulation of Rrp7 in the *GAL::RRP7-HTP* strain was efficiently depleted 12 h after shifting from galactose- to glucose-containing medium (Figure 7A). Sucrose gradient sedimentation analysis showed that depletion of Rrp7 led to the disappearance of free 40S peak but did not affect the distribution of U3 in large preribosomes (Figure 7B,C), consistent with the previous results [24]. In normal cells, only a small fraction of snR30 cosediments with large preribosomes [41],[44]. Depletion of Rrp7 caused no detectable change of the sedimentation profile of snR30, suggesting that Rrp7 does not control the association or dissociation of snR30. The distribution of snR10, which Rrp7 crosslinks, was not altered either in the absence of Rrp7.

Next, we examined whether snR30 affects the association of Rrp7 with preribosomes. To this end, the chromosomal *SNR30* gene in the *RRP7-HTP* strain was placed under the control of the *GAL1* promoter. The expression of snR30 can be efficiently repressed in glucose medium (Figure 7D). In wild-type cells, majority of Rrp7 was distributed in large particles. Upon depletion of snR30, Rrp7 was still distributed in large particles but an increase in free protein fractions was observed, suggesting that snR30 may affect the strength or dynamics of Rrp7 binding to preribosomes. In addition, the depletion of snR30 seemed not to change the distribution of snR10 in sucrose gradients.

To directly analyze the association of Rrp7 with preribosomes, we determined RNA species coimmunoprecipitated with Rrp7-HTP. The 90S preribosome could contain 35S or 23S pre-rRNA; 23S pre-rRNA is resulted from cleavage at site A3 of 35S pre-rRNA without prior cleavage at sites A0, A1, and A2 (Figure 7G). Immunoprecipitation of Rrp7-HTP coprecipitated much less U3 snoRNA and 35S/23S pre-rRNA in the absence of snR30 than in the presence of snR30 (Figure 7H). This indicates that snR30 is required for the stable incorporation of Rrp7 into preribosomes.

## Discussion

We have conducted a comprehensive structure-function analysis of Utp22 and Rrp7 and illustrated the way by which they assemble into the 90S preribosome. The complex structure of Utp22 and Rrp7 shows that they are unlikely to possess any enzymatic activity and that they rather function as an essential building block in the 90S preribosome. The binding interface between Utp22 and Rrp7 is so extensive that disruption of the intermolecular interaction at the NTD or the linker region of Rrp7 is well tolerated in vivo (Figure 4). The two proteins most likely function as a single stable module during association and dissociation with preribosomes.

We demonstrate that Rrp7 is an RNA-binding protein and efficiently crosslinks 18S rRNA. Somewhat surprisingly, the RNA-binding activity of Rrp7 is principally located on the CTD, not the RRM-like NTD. The CTD deletion mutant of Rrp7 failed to associate with preribosomes, even though it can still bind Utp22 in this case. We can infer that Utp22 cannot assemble into preribosomes on its own and should depend on Rrp7 for assembly. The CTD of Rrp7 serves as the primary anchor of the Utp22/Rrp7 complex on preribosomes.

Our NMR analysis reveals that the highly conserved C-terminal 40 residues of Rrp7 form two flexible α-helices. Such a structure is reminiscent of long tails present in many r-proteins, which are flexible in isolation yet contact rRNA over a long distance once assembled into the ribosome. We speculate that the CTD of Rrp7 could assume a similar mode during rRNA binding. While our data suggest that the CTD of Rrp7 anchors the Utp22/Rrp7 complex on 90S preribosomes through binding RNA, we cannot exclude other mechanisms, such as protein interaction, are involved.

Once assembled into the 90S preribosome, the Utp22/Rrp7 complex is expected to make contact with neighboring RNAs and proteins. Our mutagenesis data suggest that the NTD of Rrp7 and the conserved surface patches on D2 and D4 of Utp22 may be involved in interaction with other molecules to maintain a functional conformation of the 90S preribosome. In the absence of the essential NTD, Rrp7 can assemble into preribosomes but appears not to dissociate (Figure 4G). In this case, maturation of the defective 90S preribosome may be inhibited, subsequently blocking the release of assembly factors.

### A Network of Ribosome Synthesis Factors Associated Around the Central Domain of 18S rRNA

The central domain of 18S rRNA consists of helices h19–h26 and ES6. ES6 is the largest eukaryotic-specific extension segment in 18S rRNA and composed of five helices named A to E. In the 40S structure, helices h19–h26, together with the 3′-end region of 18S rRNA, make up the platform, whereas ES6 lies over the solvent side of the body (Figure 6C,E) [1],[2].

Our observation that Rrp7 binds to ES6E and h26 in the central domain of 18S rRNA is correlated with several previous genetic and biochemical results (Figure 6G). The r-protein S27 was found to be a high copy suppressor of the lethal phenotype of *rrp7* deletion [35]. In the 40S structure, S27 binds h26 adjacent to the Rrp7 crosslinking site, corroborating the genetic interaction between S27 and Rrp7. In addition, depletion of two r-proteins S13 and S14, which bind to the platform, reduced the association of Utp22 and Rrp7, among other proteins, with 90S preribosomes [45]. S13 contacts S27 in the 40S structure and is also close to the Rrp7 crosslinking sites on ES6E and h26, whereas S14 binds at one edge of the platform. S13, S14, and S27 are all required for early processing of 18S pre-rRNA [46], and they may assemble together with Utp22/Rrp7 and other ribosome biogenesis factors around the central domain to form a structural module in 90S preribosomes.

We find that the major binding region of Rrp7 on ES6E is flanked by two snR30-binding sites: rm1 and rm2. The middle sequence between rm1 and rm2 is predicted to adopt a hairpin when snR30 is bound to 18S rRNA (Figure 6D) [41]. However, in the mature 40S structure, rm1 is part of helix C of ES6, rm2 forms a long-range base-pairing interaction with ES3 at the left foot, and the middle region is unpaired or comprises one strand of the ES6E helix (Figure 6C). Apparently, dramatic structural changes should occur when the ES6E region is transformed from the snR30-bound state to the mature state. Which state of ES6E is recognized by Rrp7 is unknown. Given that snR30 is required for the stably association of Rrp7 to preribosomes but not vice versa (Figure 7), Rrp7 might be recruited downstream of snR30 and recognize the intermediate snR30-bound hairpin structure of ES6E.

Among four processing snoRNAs present in yeast, snR10 is the only one that still has an unknown binding site in preribosome [16],[17]. Our finding of Rrp7 crosslinking snR10 provides the first glimpse into the location of snR10 in preribosomes. Rrp7 crosslinks with the 3′-hairpin of snR10 (Figure S4D,E), however the function of snR10 3′-hairpin remains uncertain [16],[47]. The interaction between Rrp7 and snR10 is also supported by their genetic interaction with a common factor, Rrp5 (Figure 6G). Mutations of Rrp5 displayed a synthetic lethal phenotype with snR10 deletion [48], and snR10 is a high-dose suppressor of an Rrp5 mutant [47]. Moreover, incorporation of Rrp7 in preribosome was found dependent on prior association of Rrp5 [24].

Our data suggest that Rrp7 is located near to two processing H/ACA snoRNAs, snR30 and snR10, in preribosomes. To provide insight into other factors that are potentially associated with them around the central domain of 18S rRNA, we complied from the literature an interaction network map focused on the three molecules (Figure 6G). In addition to the interactions and factors discussed above, the map also includes Utp23, Kri1, and Rok1. Utp23 and Kri1 are two early-acting SSU synthesis factors that bind the snR30 snoRNP [49]–[51]. Rok1, an essential RNA helicase, was identified in a synthetic lethal screen with snR10 deletion [52]. Rok1 is involved in release of snR30 [53] and is a high copy suppressor of an Rrp5 mutant [54]. In this map, snR30 plays a key role in preribosome assembly since it is required for assembly of Utp23, Kri1, and Rrp7 ([49], this work) and the formation of a compact 90S particle at the terminus of nascent rRNAs [44].

Eukaryotic rRNAs contain many extension segments that contribute to increased structural complexity of eukaryotic ribosomes. The exact function of extension segments is elusive in most cases. The interaction of ES6E with snR30 [41] and Rrp7 shows that extension segments can play a role in binding ribosome synthesis factors. Another example is provided by recent cryo-EM structures of late pre-60S particles, which show that extension segment 27 of 25S rRNA interacts with the nuclear export factor Arx1 [30],[31]. The interaction between rRNA extension segments and ribosome synthesis factors illustrates that the structure of eukaryotic ribosome coevolved with its assembly machinery.

### Evolutionary Insight

The structural homology of Utp22 with dimeric class I CCA-adding enzyme is intriguing. It appears that the eukaryotic rRNA processing machinery has borrowed a factor that is involved in tRNA processing during evolution. Finding a connection between tRNA and rRNA processing machinery is, however, not unprecedented. The MRP nuclease, which is responsible for pre-rRNA cleavage at site A3 in ITS1, is homologous to RNase P, which processes the 5′-end of tRNA [55]. In addition, the catalytic subunit Cbf5 of H/ACA RNP is closely related to TruB, the synthase for tRNA pseudouridine 55 [56].

It is difficult to envision how a CCA-adding enzyme that processes tRNA evolved into an rRNA processing factor. In one scenario, the primordial eukaryotic SSU rRNA might bind a tRNA or contain a tRNA-like structure that recruits a dimeric CCA-adding enzyme. The CCA-adding enzyme might have been initially recruited for its RNA-binding property, thus allowing the unneeded polymerase active site to mutate. During the course of evolution, Utp22 recruited Rrp7 and began to rely on Rrp7 rather than its own RNA-binding ability to assemble into preribosomes. The original tRNA-binding channels of Utp22 were subsequently blocked. The D4 domain in the N-half of Utp22 remains functionally important; however, the two conserved arginine residues in Utp22 D4 do not correspond to the original tRNA-binding residues in the tail domain of CCA-adding enzyme, suggesting that D4 has a different mode in RNA binding or assumes a different function.

The Utp22 gene apparently evolved after duplication and conjugation of a class I CCA-adding enzyme gene. Notably, class I CCA-adding enzymes are specifically distributed in archaea, suggesting that Utp22 has an archaeal origin or shares a common ancestor with archaeal enzymes. In this regard, archaeal homologs have also been found for a subset of eukaryotic ribosome synthesis factors. These include RIO-type kinases, the ATPase Fab7, the RNA-binding protein Dim2/Pno1, the dimethyltransferase Dim1 (which is also present in bacteria), the nuclease Nob1, the RNA methyltransferase Emg1, and Brix domain proteins (Imp4, Ssf1, Rpf1, Rpf2, and Brx1), many of which function at late stages of 40S synthesis. In addition, H/ACA RNPs and C/D RNPs are conserved in archaea. They direct rRNA modification but are not known to mediate rRNA processing in archaea. The archaeal origin of Utp22 supports the notion that the eukaryotic ribosome synthesis machinery evolved from an archaeal-like system.

## Materials and Methods

### DNA Cloning and Protein Purification

Utp22 and Rrp7 were coexpressed in insect cells using the Bac-to-Bac system (Invitrogen). The Utp22 gene was amplified by PCR from yeast genomic DNA and cloned into pFastBac-1 with no tag. The Rrp7 gene was cloned similarly with an N-terminal His_6_-tag followed by a PreScission cleavage site. The recombinant viruses were generated in SF21 cells according to the manufacturer's instruction. For coexpression of Utp22 and Rrp7, High Five cells were cultured in SF-900 II SFM medium at 27°C to a density of 2×10^6^ cells/ml and coinfected by viruses expressing each protein for 48–60 h. Cells were harvested from 1 L medium and resuspended in 100 ml of lysis buffer (50 mM Tris, pH 8.0, 500 mM NaCl, 30 mM imidazole, 5% glycerol, and 2 mM β-mercaptoethanol). The sample was supplemented with two complete, EDTA-free protease inhibitor cocktail tablets (Roche) and lysed by sonication. After centrifugation at 200,000 *g*, the supernatant was loaded onto a 5-ml HisTrap column (GE Healthcare). After washing with lysis buffer, the protein was eluted with a linear gradient of imidazole. The combined fractions were diluted 3-fold with buffer A (50 mM Tris, pH 8.0, 5% glycerol) and incubated with PreScission protease overnight at 4°C to cleave the His_6_-tag from Rrp7. The protein was loaded onto a heparin column, washed with 500 mM NaCl, and eluted with 725 mM NaCl in buffer A. The protein was further purified with a HiLoad 16/60 Superdex 200 column using buffer 10 mM Tris (pH 8.0) and 200 mM NaCl, and then concentrated to 6.5 mg/ml for crystallization.

For selenomethionine labeling, the infected cells were spun down 8 h postinfection and resuspended in 1 L of SF-900 II methionine-free, cystine-free SFM media supplemented with 200 mg/L L-cysteine. The cells were cultured for 8 h, supplemented with 250 mg selenomethionine per liter, and harvested after an additional 36 h of growth. The labeled protein was purified in the same way as the unlabeled protein.

For purification of Utp22 alone, Utp22 was fused with an N-terminal noncleavable His_6_-tag. Utp22 was expressed and purified in the same way as the Utp22/Rrp7 complex. The Rrp7 protein and its fragments were expressed in *E. coli*. Rrp7 and its fragments were cloned into the plasmid pETDuet-1 and fused to an N-terminal His_6_-tag, the SMT3 protein, and a PreScission cleavage site. The protein was induced for expression in the Rosetta (DE3) strain using 0.1 mM IPTG for 16 h at 16°C. The cells were resuspended in buffer containing 50 mM Tris, pH 8.0, 300 mM NaCl, 5% glycerol, and 30 mM imidazole, which was supplemented with 100 µM phenylmethylsulfonyl fluoride and disrupted using a high-pressure cell disruptor (JNBIO). After clarification, the supernatant was applied to a 5-ml HisTrap column and the protein was eluted with imidazole. The N-terminal His_6_-tag and the SMT3 fusion protein were removed by overnight PreScission digestion at 4°C. The protein was further purified through a heparin column and a gel filtration column equilibrated in 10 mM Tris, pH 7.5, 200 mM NaCl. For NMR study, Rrp7 256–297 was labeled with ^15^N and ^13^C in M9 minimal medium containing 1 g/L of (^15^NH_4_)_2_SO_4_ and 2 g/L of ^13^C-glucose (Cambridge Isotope Laboratories).

### Crystallization and Structure Determination

Crystals of the Utp22 and Rrp7 complex (6.5 mg/ml in 10 mM Tris, pH 8.0, and 200 mM NaCl) were grown from 100 mM sodium cacodylate pH 6.2–6.5, 30% (w/v) PEG 400, and 200 mM lithium sulfate by hanging drop vapor diffusion method at 20°C and were directly frozen in liquid nitrogen without further cryoprotection. The Se-labeled protein was purified and crystallized in the same way as the native protein. A Se-derivative dataset was collected to 3.0 Å resolution at beamline BL17U of the Shanghai Synchrotron Radiation Facility, processed with HKL2000 [57], and used for SAD phasing in SHARP [58]. After density modification, the electron density map was of sufficient quality to allow automatic model building in ARP/wARP [59]. The model was further adjusted in Coot [60] and refined with PHENIX and refmac [61],[62]. A native dataset was collected at Japan SPring-8 beamline BL41XU and used for final refinement at 1.97 Å resolution. The current model contains Utp22 residues 81–274, 282–317, 326–445, 453–983, 1010–1116, and 1128–1237; Rrp7 residues 3–27, 32–105, and 120–189; 764 water molecules; 11 sulfate ions; and three PEG molecules. Analysis with RAMPAGE showed that 98.5% of the residues are in favored regions, 1.4% are in allowed regions, and 0.1% are in outlier regions. Structural figures were prepared using PyMOL [63].

### NMR Structure Determination of Rrp7 256–297

The NMR sample contained 1.0 mM ^15^N/^13^C-labeled Rrp7 256–297, 50 mM potassium phosphate (pH 6.0), and 10% (v/v) ^2^H_2_O. NMR spectra were measured at 298 K on a Bruker DMX600 spectrometer equipped with a triple resonance cryoprobe. Spectra ^1^H-^15^N HSQC, ^1^H-^15^N TOCSY-HSQC, CBCA(CO)NH, HNCACB, HNCO, HN(CA)CO, HBHA(CBCA)(CO)NH, HBHA(CBCA)NH, CCH-TOCSY, and (H)CCH-TOCSY were collected and used to obtain backbone and side chain resonance assignments. Spectra were processed with Felix (Accelrys Inc.) and analyzed with NMRViewJ [64]. 3D ^1^H-^15^N NOESY-HSQC (τ_m_ 200 ms) and 3D aliphatic ^1^H-^13^C NOESY-HSQC (τ_m_ 200 ms) spectra were recorded to derive NOE distance restraints. Backbone dihedral angle restraints were calculated by analyzing HN, Hα, Cα, Cβ, C′, and N chemical shifts in TALOS+ [65]. The structure was calculated in CYANA and further refined in CNS by incorporating additional dihedral angle restraints [66],[67]. The 20 lowest energy structures out of 100 calculated structures were analyzed.

### EMSA

snR5 RNA was in vitro transcribed, dephosphorylated, labeled with ^32^P at the 5′-end, and column-purified using standard methods. Approximately 0.1 nM labeled RNA was incubated with protein in a 10 µl reaction containing 25 mM HEPES-K (pH 7.6), 100 mM NaCl, 2 mM MgCl_2_, 1 mM DTT, 0.01% NP-40, and 10% glycerol at room temperature for 10 min. The reactions were resolved in 5% native polyacrylamide gels running in 1× Tris-glycine (pH 8.3) buffer at room temperature. The gels were dried and autoradiographed using a Typhoon PhosphorImager (GE Healthcare).

### Yeast Strains, Media, Plasmids, and Cloning

Yeast cells were grown in YPDA (1% yeast extract, 2% peptone, 0.003% adenine, and 2% glucose), YPGA (1% yeast extract, 2% peptone, 0.003% adenine, and 2% galactose), Synthetic Complete (SC) medium, and appropriate SC dropout medium (Clontech). Yeast cells were transformed using the lithium acetate method.

Gene cloning was mainly preformed using the non-ligation-based In-fusion (TaKaRa) or Transfer-PCR approaches [68]. Mutagenesis was conducted with QuikChange. All plasmids were verified by DNA sequencing. The strains, primers, and plasmids generated are listed in Tables S4, S5, and S6.

Chromosomal tagging was performed using the one-step PCR strategy. The *GAL1* promoter cassette was amplified from plasmid pFA6a–His3MX6–PGAL1 [69]. To generate the *RRP7* shuffle strain, the heterozygous deletion diploid *rrp7Δ/RRP7* (Euroscarf) was transformed with a *URA3* pRS416 plasmid carrying *RRP7* under its endogenous promoter. The transformants were sporulated, and isolated spores were germinated to select for the *rrp7Δ* haploid complemented with the *URA3 RRP7* plasmid in Ura-deficient SC medium containing G418. The *UTP22* shuffle strain was generated in a similar manner from the *utp22Δ/UTP22* strain.

To construct a HTP cassette for genomic tagging, the ProtA–TEV–His_7_ tag in plasmid pYM9 [70] was modified to remove the original His-tag and incorporate a new His_7_-tag before Protein A, yielding plasmid pYM9–HTP. The *RRP7*–*HTP* and *UTP22*–*HTP* strains were generated by integrating the HTP cassette into strain BY4741.

### Spot Assay

Yeast cells were inoculated into 2 ml of YPDA liquid medium and cultured at 30°C until OD_600_ reached 0.6–1.0. The culture was adjusted to OD_600 = _0.6 and serially diluted 10-fold with sterile water. The sample was spotted on plates containing SC medium with or without 0.1% 5-FOA and incubated at 37, 30, and 20°C for 4 d.

### Sucrose Gradient Sedimentation

To deplete *GAL*-driven genes, logarithmically growing cells (OD_600_ = 0.6–1.0) cultured in YPGA medium were harvested, washed with water, and re-suspended in YPDA medium. The *GAL::SNR30* strain was grown in YPDA medium for 14 h and the *GAL::RRP7-HTP* strain was grown in YPDA medium for 16 h.

Polysome profile analysis was preformed as previously described [71]. Yeast cells (250–300 ml) were grown to OD_600 = _0.8–1.0 and supplied with 0.1 mg/ml of cycloheximide (Sigma) immediately before harvesting. Pelleted cells were resuspended in 500 µl of lysis buffer (10 mM Tris, pH 7.5, 100 mM NaCl, 30 mM MgCl_2_, 0.1 mg/ml cycloheximide, and 0.2 mg/ml heparin) and lysed by vortexing with acid-washed, baked glass beads. After clarification by centrifugation at 15,000 *g* for 10 min at 4°C, 350 µl of extracts equivalent to 15–20 OD_260_ units were layered onto a 10 ml 7–50% sucrose gradient prepared in 50 mM Tris-acetate, pH 7.5, 50 mM NH_4_Cl, 12 mM MgCl_2_, and 1 mM DTT. Samples were centrifuged in a SW41 Ti rotor (Beckman) at 39,000 rpm at 4°C for 165 min. Gradients were manually fractionated in ∼0.5 ml volume using a gradient collector (ISCO). Ribosome profiles were recorded by measuring UV absorbance at 254 nm. Proteins from 20 µl of fractions were separated by SDS-PAGE and analyzed with Western blotting. RNA was extracted from 100 µl of gradient fractions and analyzed with Northern blotting.

### Immunoprecipitation

Yeast cells were lysed using glass beads in lysis buffer containing 20 mM Tris-HCl (pH 8.0), 5 mM Mg-acetate, 10 mM NaCl, and 0.2% Triton X-100, supplemented with one tablet of EDTA-free protease inhibitor cocktail (Roche), 0.5 U/µl RNasin (Promega), and 1 mM DTT. After clarification by centrifugation, IgG Sepharose beads (100 µl) were incubated with 100 OD_260_ units of supernatant for 2 h and washed seven times with 800 µl of lysis buffer containing 200 mM NaCl. Twenty percent of the beads were used for protein analysis, and the remaining beads were used for RNA extraction.

### Western Blot Analysis

Proteins were separated in 12% SDS-PAGE gels and transferred to nitrocellulose membranes (Whatman) or PVDF membranes (GE Healthcare) using a semi-dry electrophoretic transfer cell (BioRad). The following primary antibodies were used with appropriate dilution ratios: peroxidase anti-peroxidase (1∶5,000, Sigma) and anti-DYKDDDK tag mouse antibody (1∶5,000, Abmart). The secondary antibody used was sheep anti-mouse IgG-horseradish peroxidase (1∶5,000, GE Healthcare).

### Northern Blot Analysis

RNA was isolated using TRIzol reagent (Invitrogen) or the hot phenol method. High molecular weight RNAs were separated in 1.2% agarose-formaldehyde gels, and low molecular weight RNAs were separated in 8% polyacrylamide–8 M urea gels. RNAs were transferred to Hybond N^+^ membranes (GE Healthcare). The following oligonucleotides were used for northern hybridization: D-A2: 5′-CGGTTTTAATTGTCCTA; snR30: 5′-ATGTCTGCAGTATGGTTTTAC; U3: 5′-GGATTGCGGACCAAGCTAA; snR10: 5′-GTGTTACGAATGGCTGTTA. Oligonucleotides were 5′-end labeled with [γ-^32^P] ATP using T4 polynucleotide kinase (New England Biolab) and purified using MicroSpin G-25 columns (GE Healthcare). Prehybridization and hybridization were preformed in PerfectHyb Plus hybridization buffer (Sigma). After washing once in 2×SSC (300 mM NaCl, 30 mM sodium-citrate) including 0.1% SDS and twice in 1×SSC including 0.5% SDS, membranes were visualized by phosphorimaging or X-ray film exposure.

### Yeast Two-Hybrid Assay

Two-hybrid assays were performed using the MATCHMAKER GAL4 two-hybrid system (Clontech). Utp22 was cloned into the GAL4 DNA-binding domain (BD) vector pGBKT7 as bait. Rrp7 was cloned into the GAL4 DNA activation domain (AD) vector pGADT7 as prey. The two plasmids were co-transformed into strain AH109, which expresses the *HIS3* and *ADE2* reporter genes under the control of the *GAL4* promoter. The Leu^+^ Trp^+^ transformants were grown in 3 ml of SC medium lacking Leu and Trp overnight at 30°C and adjusted to an OD_600_ of 0.6. The cells were 10-fold serially diluted with water and spotted on plates with SC medium lacking Leu and Trp; on SC medium lacking Leu, Trp, and His and containing 5 mM of 3-amino-1,2,4-triazole (3-AT); or on SC medium lacking Leu, Trp, His, and Ade. The plates were incubated for 3 d at 30°C.

### CRAC

CRAC experiments were performed as previously described with the following changes [27]. Briefly, yeast cells were grown from 1 L YPDA medium to OD_600_ ∼0.5 (1.5 g), UV crosslinked in Petri dishes with a Stratalinker (Stratagene), and lysed with glass beads. HTP-tagged proteins were bound to IgG Sepharose beads and eluted after TEV cleavage. The samples were incubated with RNase A/T1 mixture for 10 min at 37°C to partially digest crosslinked RNA. Guanidine-HCl was added to 6 M and the samples were then bound to MagnetHis Ni-Particles (Promega). Crosslinked RNAs were dephosphorylated, ligated to the 3′ linker and 5′-end ^32^P-labeled on beads. The 5′-linker was not ligated at this step. Proteins were eluted with imidazole, resolved in Bis-Tris NuPAGE gels (Invitrogen), and blotted onto nitrocellulose membranes. After exposure to X-ray film, the radioactive RNP band was excised, sliced, and incubated with proteinase K. The released RNA was purified by phenol extraction and ethanol precipitation and ligated to the 5′ linker. The ligation reaction was resolved in a 20% polyacrylamide/8 M urea gel. The gel band containing radioactive RNA was excised and crushed. RNA was soaked out in 0.4 M NaCl overnight at 4°C, filtered through a Costar Spin-X column (Sigma), and ethanol precipitated. cDNA was synthesized by reverse transcription using the primer DP3 and amplified by PCR (25–35 cycles) using the primers DP3 and DP5 (Table S7). PCR products were resolved in a 10% denaturing polyacrylamide gel, and DNA of expected size was purified using a QIAEX II kit (Qiagen). For Sanger sequencing, DNA was cloned into the pCR4-topo vector. For Solexa sequencing, 2 µl of the first PCR product was PCR-amplified (6–14 cycles) using the primers SBS3 and SBS5 (Table S7). The second PCR product was purified in 3% MetaPhor agarose gels (Lonza) and sent for deep sequencing (Illumina).

Two million reads were aligned to the yeast genomic reference sequence Saccharomyces_cerevisiae.EF2.59.1.0 using the free version of Novoalign 2.08 (Novocraft). The alignment was analyzed using the pyCRAC 1.0.3.2 tool suite (Sander Granneman, unpublished).

### Accession Numbers

The atomic coordinates and experimental data have been deposited in the Protein Data Bank under accession codes 4M5D for the Utp22 and Rrp7 complex and 2MBY for Rrp7 256–297. The NMR resonance assignments for Rrp7 256–297 have been deposited in BioMagResBank under accession number 19416.

## Supporting Information

Figure S1 **Structural comparison of Utp22 and AfCCA.** (A–B) The combined D1–D2 (A) or D5–D6 (B) domain of Utp22 is aligned to the combined head and neck domain of AfCCA. The RMSD values are 1.675 Å for 104 Cα atoms of D1–D2 and 1.535 Å for 74 Cα atoms of D5–D6. In the Utp22 structure, D1 and D5 are shown in magenta and D2 and D6 are shown in cyan. The AfCCA tRNA complex structure is shown in grey. In the active site of AfCCA, a bound ATP is shown as red sticks, a Mg^2+^ ion as a green sphere, and three catalytic acidic residues as silver sticks. Utp22 residues that are equivalent to three catalytic residues of AfCCA are shown as sticks and labeled. (C–D) The D3 (C) or D7 (D) domain of Utp22 is aligned to the body domain of AfCCA. The RMSD values are 1.640 Å for 26 Cα atoms of D3 and 2.053 Å for 17 Cα atoms of D7. The D3 and D7 domains of Utp22 are shown in green and the D4 and D8 domains are shown in yellow. AfCCA is shown in grey. (E) Structural comparison of Utp22 with AfCCA dimer. The two structures are oriented such that the dyad or pseudo-dyad axis (shown as ellipse) of each structure is perpendicular to the paper. The equivalent domains are shown in the same color.
(TIF)

Figure S2 **NMR structure of Rrp7 256–297.** (A) ^1^H-^15^N HSQC spectrum of Rrp7 256–297. The spectrum was collected with 1.0 mM ^15^N/^13^C-labeled Rrp7 256–297 in 50 mM potassium phosphate (pH 6.0) and 10% (v/v) ^2^H_2_O at 298 K. The residue numbers of assigned peaks are indicated. Amide protons from the same Asn or Gln side chain are connected by lines. Residues 252–255 (GPEA) are from the cloning vector. (B–C) The Cα traces of the 20 lowest energy structures are aligned by helices α5 (B) or α6 (C). The orientation between the two helices is not fixed.
(TIF)

Figure S3 **Dominant negative effect of Rrp7 Δ190–297.** (A) Ribosomal profiles of sucrose gradient sedimentation. Extracts of *RRP7–HTP* cells expressing pGAL–RRP7 or pGAL–RRP7 Δ190–297 and grown in galactose were analyzed with 7–50% sucrose gradients. Ribosomal sedimentation profiles were recorded by measuring the absorbance at 254 nm. (B) The BY4741 strain was transformed with an empty 2 µ plasmid, pGAL–RRP7, pGAL–RRP7 Δ190–297, pGAL–RRP7 Δ1–89, or pGAL–RRP7 Δ1–156, diluted in a 10-fold series, and spotted onto plates containing glucose (Glu) or galactose (Gal) media. The plates were incubated for 3 d at 37°C, for 3 d at 30°C, and for 5 d at 20°C.
(TIF)

Figure S4 **RNA crosslinking sites of Rrp7.** (A) Distribution of nucleotide mutations and deletions in RNA reads mapped to the 18S ES6E and h26 regions. (B–C) Alignment of randomly selected deletion-containing reads to the ES6E (D) and h27 (E) regions of 18S. The 18S rRNA sequence is shown on the top. Frequent deletion sites are marked with asterisks. (D) Crosslinking of Rrp7 with snR10. The number of hits from 2 million reads mapped to each nucleotide of snR10 is plotted as a black line using the left *y*-axis scale. The number of mutations (red) and deletions (green) in mapped reads are plotted using the right *y*-axis scale. (E) Secondary structure of snR10. Rrp7 crosslinking sites are shaded in green. The pseudouridylation substrate RNA of 25S for the 3′-hairpin is shown.
(TIF)

Table S1 **Data collection and refinement statistics of the crystal structure of Utp22 and Rrp7 complex.**
(DOC)

Table S2 **NMR structure determination statistics for Rrp7 256–297.**
(DOC)

Table S3 **RNA crosslinking hits of Rrp7.**
(DOC)

Table S4 **Strain list.**
(DOC)

Table S5 **Oligonucleotide list.**
(DOC)

Table S6 **Plasmid list.**
(DOC)

Table S7 **Oligonucleotides for CRAC.**
(DOC)

Text S1 **Alignment of 151 Utp22 sequences. The alignment file is in the fasta format.**
(FASTA)

Text S2 **Alignment of 115 Rrp7 sequences. The alignment file is in the fasta format.**
(FASTA)

## Abbreviations

CRAC: UV crosslinking and analysis of cDNA
CTD: C-terminal domain
EMSA: electrophoretic mobility shift assay
ES: extension segment
HTP: His_7_–TEV–Protein A
LSU: large subunit
NOE: nuclear Overhauser effect
NTD: N-terminal domain
pre-rRNA: precursor ribosomal ribonucleic acid
RNP: ribonucleoprotein particle
r-protein: ribosomal protein
RRM: RNA-recognition motif
rRNA: ribosomal ribonucleic acid
snoRNA: small nucleolar RNA
SSU: small subunit
