# Supplementary figures and images for: An RNA-Binding Complex Involved in Ribosome Biogenesis Contains a Protein with Homology to tRNA CCA-Adding Enzyme
Source: PLoS Biol. 2013 Oct 1;11(10):e1001669. doi: 10.1371/journal.pbio.1001669 (PMC3794860; doi:10.1371/journal.pbio.1001669)

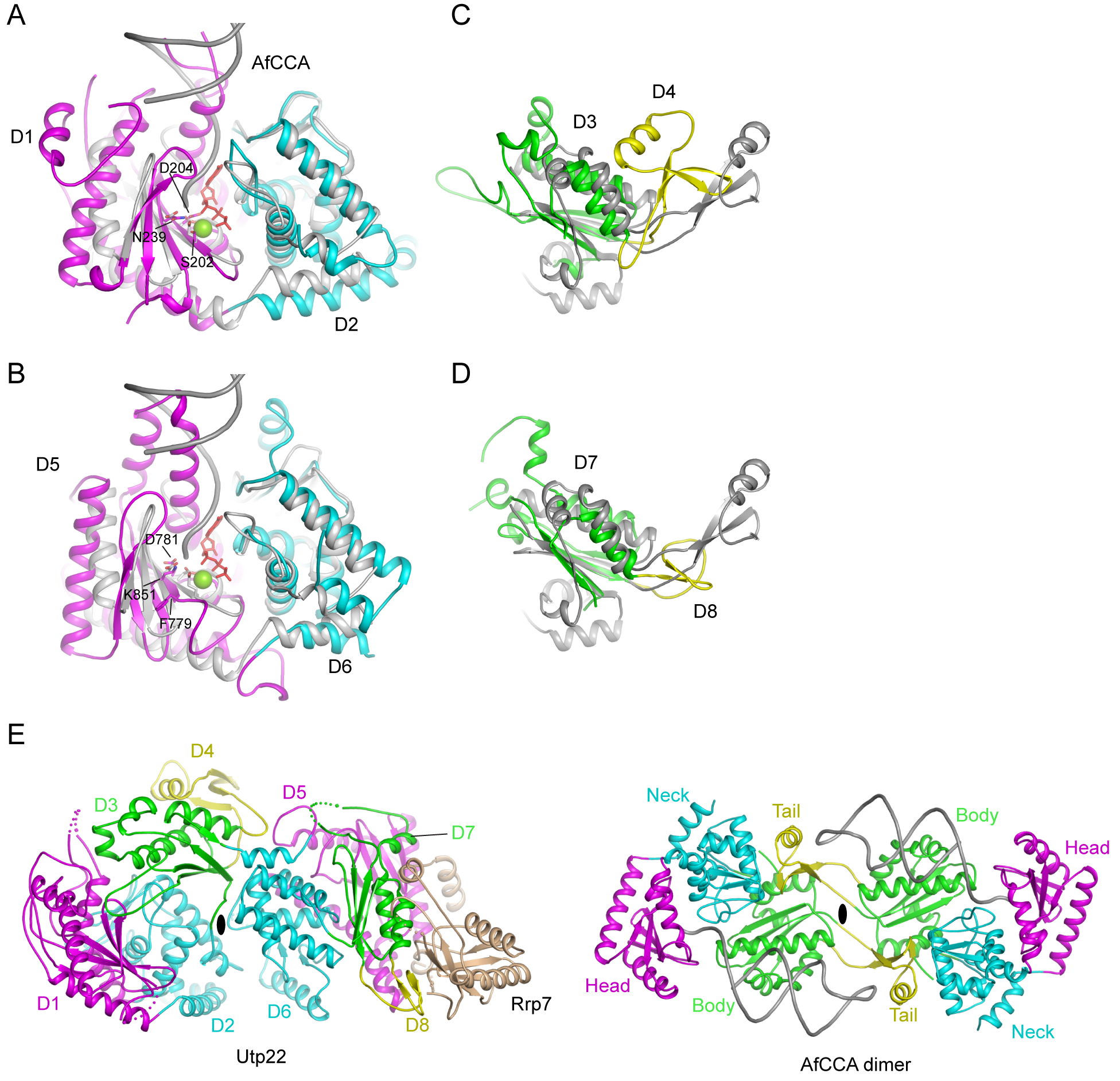

Supplement: Figure S1 — Structural comparison of Utp22 and AfCCA. (A–B) The combined D1–D2 (A) or D5–D6 (B) domain of Utp22 is aligned to the combined head and neck domain of AfCCA. The RMSD values are 1.675 Å for 104 Cα atoms of D1–D2 and 1.535 Å for 74 Cα atoms of D5–D6. In the Utp22 structure, D1 and D5 are shown in magenta and D2 and D6 are shown in cyan. The AfCCA tRNA complex structure is shown in grey. In the active site of AfCCA, a bound ATP is shown as red sticks, a Mg2+ ion as a green sphere, and three catalytic acidic residues as silver sticks. Utp22 residues that are equivalent to three catalytic residues of AfCCA are shown as sticks and labeled. (C–D) The D3 (C) or D7 (D) domain of Utp22 is aligned to the body domain of AfCCA. The RMSD values are 1.640 Å for 26 Cα atoms of D3 and 2.053 Å for 17 Cα atoms of D7. The D3 and D7 domains of Utp22 are shown in green and the D4 and D8 domains are shown in yellow. AfCCA is shown in grey. (E) Structural comparison of Utp22 with AfCCA dimer. The two structures are oriented such that the dyad or pseudo-dyad axis (shown as ellipse) of each structure is perpendicular to the paper. The equivalent domains are shown in the same color. (TIF) [file pbio.1001669.s001.tif]

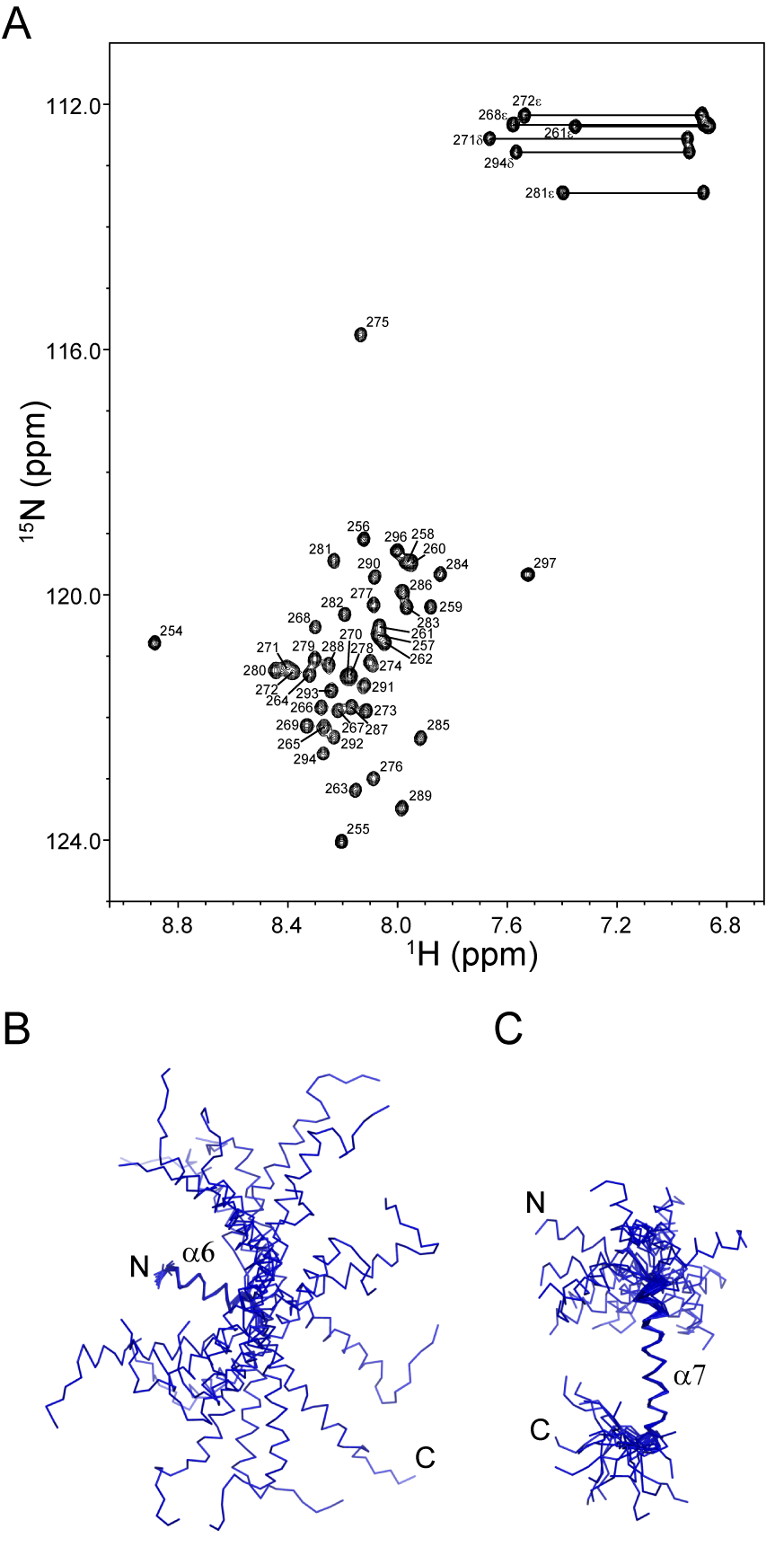

Supplement: Figure S2 — NMR structure of Rrp7 256–297. (A) 1H-15N HSQC spectrum of Rrp7 256–297. The spectrum was collected with 1.0 mM 15N/13C-labeled Rrp7 256–297 in 50 mM potassium phosphate (pH 6.0) and 10% (v/v) 2H2O at 298 K. The residue numbers of assigned peaks are indicated. Amide protons from the same Asn or Gln side chain are connected by lines. Residues 252–255 (GPEA) are from the cloning vector. (B–C) The Cα traces of the 20 lowest energy structures are aligned by helices α5 (B) or α6 (C). The orientation between the two helices is not fixed. (TIF) [file pbio.1001669.s002.tif]

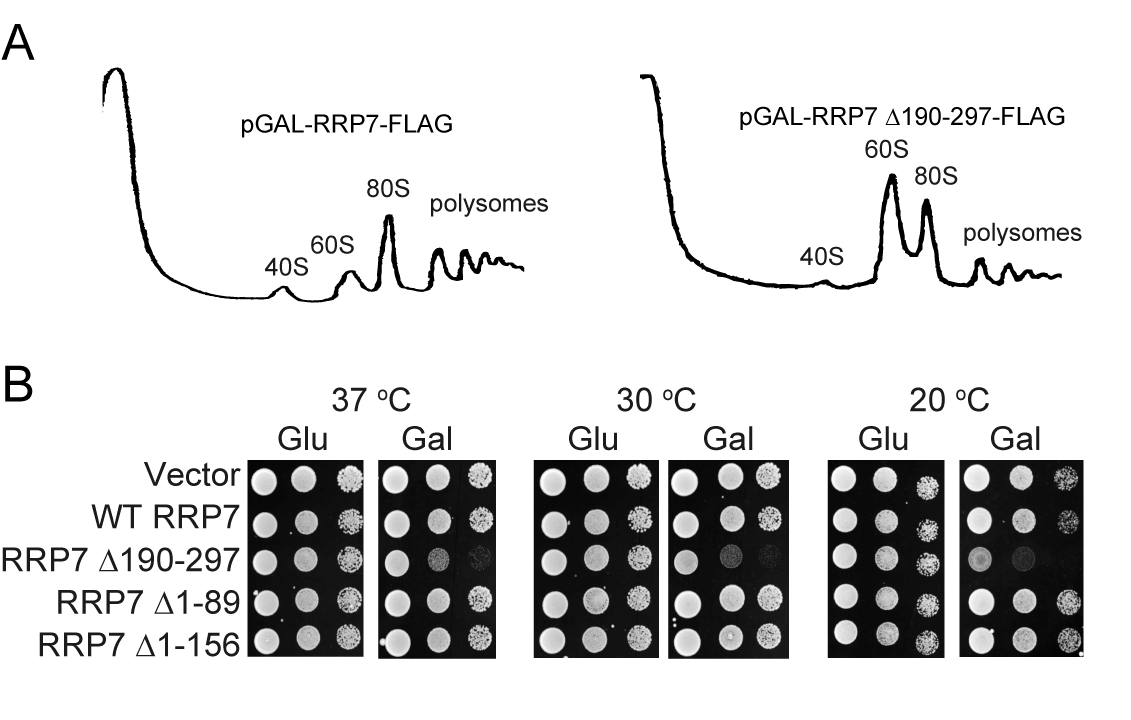

Supplement: Figure S3 — Dominant negative effect of Rrp7 Δ190–297. (A) Ribosomal profiles of sucrose gradient sedimentation. Extracts of RRP7–HTP cells expressing pGAL–RRP7 or pGAL–RRP7 Δ190–297 and grown in galactose were analyzed with 7–50% sucrose gradients. Ribosomal sedimentation profiles were recorded by measuring the absorbance at 254 nm. (B) The BY4741 strain was transformed with an empty 2 µ plasmid, pGAL–RRP7, pGAL–RRP7 Δ190–297, pGAL–RRP7 Δ1–89, or pGAL–RRP7 Δ1–156, diluted in a 10-fold series, and spotted onto plates containing glucose (Glu) or galactose (Gal) media. The plates were incubated for 3 d at 37°C, for 3 d at 30°C, and for 5 d at 20°C. (TIF) [file pbio.1001669.s003.tif]

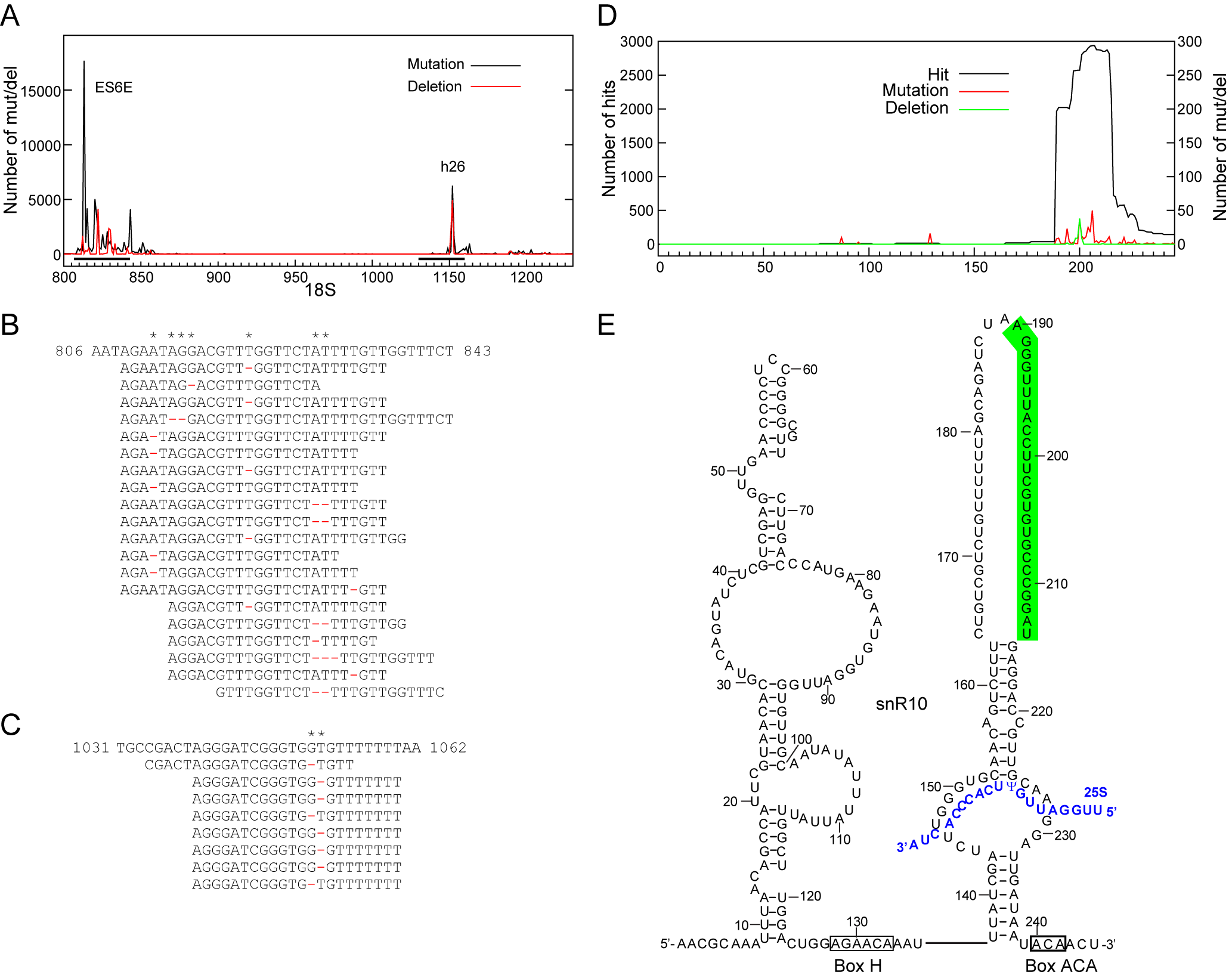

Supplement: Figure S4 — RNA crosslinking sites of Rrp7. (A) Distribution of nucleotide mutations and deletions in RNA reads mapped to the 18S ES6E and h26 regions. (B–C) Alignment of randomly selected deletion-containing reads to the ES6E (D) and h27 (E) regions of 18S. The 18S rRNA sequence is shown on the top. Frequent deletion sites are marked with asterisks. (D) Crosslinking of Rrp7 with snR10. The number of hits from 2 million reads mapped to each nucleotide of snR10 is plotted as a black line using the left y-axis scale. The number of mutations (red) and deletions (green) in mapped reads are plotted using the right y-axis scale. (E) Secondary structure of snR10. Rrp7 crosslinking sites are shaded in green. The pseudouridylation substrate RNA of 25S for the 3′-hairpin is shown. (TIF) [file pbio.1001669.s004.tif]
