# Supplementary material for: An RNA-Binding Complex Involved in Ribosome Biogenesis Contains a Protein with Homology to tRNA CCA-Adding Enzyme
Source: PLoS Biol. 2013 Oct 1;11(10):e1001669. doi: 10.1371/journal.pbio.1001669 (PMC3794860; doi:10.1371/journal.pbio.1001669)
Supplement: Table S1 — Data collection and refinement statistics of the crystal structure of Utp22 and Rrp7 complex. (DOC) [file pbio.1001669.s005.doc]

Table S1. Data collection and refinement statistics of the crystal structure of Utp22 and Rrp7 complex.

| Crystal form | Se-labeled | Native |
| --- | --- | --- |
| *Data collection* |  |  |
| Space group | I222 | I222 |
| Cell dimensions |  |  |
| a, b, c (Å) | 126.2, 129.5, 215.6 | 126.3, 129.6, 214.4 |
| α, β, γ (º) | 90, 90, 90 | 90, 90, 90 |
| Wavelength (Å) | 0.9796 | 1.0 |
| Resolution range (Å) | 50-3.0(3.05-3.00) | 50-1.97(2.04-1.97) |
| Unique reflections | 67918 | 123322 |
| Redundancy | 7.7 (5.9) | 4.2 (4.1) |
| <*I*>/<σ(*I*)> | 16.5 (2.3) | 15.9 (2.4) |
| Completeness (%) | 99.5 (99.4) | 99.3 (92.7) |
| *R*_merge_ | 0.153 (0.640) | 0.083 (0.577) |
| *Structure refinement* |  |  |
| Resolution range (Å) |  | 20-1.97(2.02-1.97) |
| No. of reflections |  | 116965 (8897) |
| No. of atoms |  | 11081 |
| Mean B factors(Å^2^) |  | 30.6 |
| *R*_work_ |  | 0.210(0.255) |
| *R*_free_ |  | 0.239(0.305) |
| Rmsd bond length (Å) |  | 0.010 |
| Rmsd bond angles (º) |  | 1.113 |

Values for the data in the highest resolution bin are shown in parentheses
