# Supplementary material for: An RNA-Binding Complex Involved in Ribosome Biogenesis Contains a Protein with Homology to tRNA CCA-Adding Enzyme
Source: PLoS Biol. 2013 Oct 1;11(10):e1001669. doi: 10.1371/journal.pbio.1001669 (PMC3794860; doi:10.1371/journal.pbio.1001669)
Supplement: Table S2 — NMR structure determination statistics for Rrp7 256–297. (DOC) [file pbio.1001669.s006.doc]

Table S2. NMR structure determination statistics for Rrp7 256-297.

| **Restraint statistics** |  |
| --- | --- |
| Distance restraints |  |
| Total NOE | 563 |
| Intra-residue | 297 |
| Sequential (|*i* – *j*| = 1) | 144 |
| Medium-range (|*i* – *j*| ≤4) | 122 |
| Long-range (|*i* – *j*| ≥ 5) | 0 |
| Ambiguous | 0 |
| Total dihedral angle restraints |  |
| φ | 34 |
| ψ | 34 |
| **Structure statistics** |  |
| Violations |  |
| Max. dihedral angle violation (º) | 0 |
| Max. Distance constraint violation (Å) | 0 |
| Deviations from idealized geometry |  |
| Bond lengths (Å) | 0.0013±0.00006 |
| Bond angles (º) | 0.28±0.006 |
| Impropers (º) | 0.12±0.02 |
| RMSD of all structures to mean (Å)  (residues 254-264; residues 270-288) |  |
| Heavy | 1.19±0.19; 0.87±0.14 |
| Backbone | 0.26±0.1; 0.28±0.12 |
| Ramachandran plot analysis (residues 254-264 and 270-288; all residues) |  |
| Most favored regions | 97.5%; 83.2% |
| Additional allowed regions | 2.5%; 12.9% |
| Generously allowed regions | 0%; 2.0% |
| Disallowed regions | 0%; 1.9% |
