# Supplementary material for: An RNA-Binding Complex Involved in Ribosome Biogenesis Contains a Protein with Homology to tRNA CCA-Adding Enzyme
Source: PLoS Biol. 2013 Oct 1;11(10):e1001669. doi: 10.1371/journal.pbio.1001669 (PMC3794860; doi:10.1371/journal.pbio.1001669)
Supplement: Table S3 — RNA crosslinking hits of Rrp7. (DOC) [file pbio.1001669.s007.doc]

Table S3. RNA crosslinking hits of Rrp7.

| Feature | Hits | Percentage of mapped reads |
| --- | --- | --- |
| rDNA | 1115226 | 92.75 |
| Protein coding genes | 55860 | 4.65 |
| Intergenetic regions | 13075 | 1.09 |
| snoRNAs | 5084 | 0.42 |
| tRNAs | 18797 | 1.56 |
| snRNAs | 639 | 0.05 |
| Mitochondrial rRNAs | 2201 | 0.18 |
| Other noncoding RNAs | 150 | 0.01 |
| Total mapped reads | 1202392 |  |

Two million reads were aligned to Saccharomyces_cerevisiae.EF2.59.1.0.fa by Novoalign. The resultant alignment was analyzed by pyCRAC 1.0.3.2.

Among the reads mapped to protein coding genes, 74.9% map to the antisense strand of YLR162W. YLR162W is linked to RDN5-6 in chromosome XII and the mapped region is homologous with 3'-end of 25S rRNA.
