# Supplementary material for: An RNA-Binding Complex Involved in Ribosome Biogenesis Contains a Protein with Homology to tRNA CCA-Adding Enzyme
Source: PLoS Biol. 2013 Oct 1;11(10):e1001669. doi: 10.1371/journal.pbio.1001669 (PMC3794860; doi:10.1371/journal.pbio.1001669)
Supplement: Table S4 — Strain list. (DOC) [file pbio.1001669.s008.doc]

Table S4.Strain list

| Strain | Genotype | Information |
| --- | --- | --- |
| BY4741 | *MATa, his3Δ1, leu2Δ0, met15Δ0, ura3Δ0* | Provided by Euroscarf |
| RRP7-HTP | *MATa, his3Δ1, leu2Δ0, met15Δ0, ura3Δ0, rrp7-HTP::kanMX6* | The HTP cassette was amplified from plasmid pYM9-HTP with primers Rrp7-HTP-F and Rrp7-HTP-R and integrated into the BY4741 strain using the kanMX6 selection marker. |
| Utp22-HTP | *MATa, his3Δ1, leu2Δ0, met15Δ0, ura3Δ0, utp22-HTP::kanMX6* | The HTP cassette was amplified from plasmid pYM9-HTP with primers UTP22-HTP-F and UTP22-HTP-R and integrated into the BY4741 strain using the kanMX6 selection marker. |
| GAL::RRP7-HTP | *MATa, his3Δ1, leu2Δ0, met15Δ0, ura3Δ0, His3MX6-GAL1::rrp7-HTP::kanMX6* | The GAL1 promoter cassette was amplified from plasmid pFA6a-His3MX6-PGAL1 with primers pGal-Rrp7-F and pGal-Rrp7-R and integrated into the Rrp7-HTP:: kanMX6 strain using the His3MX6 selection marker. |
| GAL::SNR30; RRP7-HTP | *MATa, his3Δ1, leu2Δ0, met15Δ0, ura3Δ0, rrp7-HTP::kanMX6, His3MX6-GAL1::snr30* | The GAL1 promoter cassette was amplified from plasmid pFA6a-His3MX6-PGAL1 with primers pGal-snR30-F and pGal-snR30-R and integrated into the Rrp7-HTP:: kanMX6 strain using the His3MX6 selection marker. |
| utp22Δ/UTP22 | *MATa/α, his3Δ1/his3Δ1, leu2Δ0/leu2Δ0, lys2Δ0/LYS2, MET15/met15Δ0, ura3Δ0/ura3Δ0, YGR090w::kanMX4/YGR090w* | Provided by Euroscarf. |
| UTP22 shuffle | *MAT a (or α), his3Δ1, leu2Δ0, lys2Δ0 (or LYS2), met15Δ0 (or MET15), ura3Δ0, YGR090w::kanMX4; [pRS416-UTP22]* | The utp22Δ/UTP22 diploid stain was transformed with pRS416-UTP22. The transformants were sporulated and isolated spores were germinated and selected in Ura-deficient SC medium containing G418. |
| rrp7Δ/RRP7 | *MAT a/α, his3Δ1/his3Δ1, leu2Δ0/leu2Δ0, lys2Δ0/LYS2, MET15/met15Δ0, ura3Δ0/ura3Δ0, YCL031c::kanMX4/YCL031c* | Provided by Euroscarf. |
| RRP7 shuffle | *MAT a (or α), his3Δ1, leu2Δ0, lys2Δ0 (or LYS2), met15Δ0 (or MET15), ura3Δ0, YCL031c::kanMX4; [pRS416-RRP7]* | The rrp7Δ/RRP7 diploid stain was transformed with pRS416-RRP7. The transformants were sporulated and isolated spores were germinated and selected in Ura-deficient SC medium containing G418. |
