# Supplementary material for: An RNA-Binding Complex Involved in Ribosome Biogenesis Contains a Protein with Homology to tRNA CCA-Adding Enzyme
Source: PLoS Biol. 2013 Oct 1;11(10):e1001669. doi: 10.1371/journal.pbio.1001669 (PMC3794860; doi:10.1371/journal.pbio.1001669)
Supplement: Table S5 — Oligonucleotide list. (DOC) [file pbio.1001669.s009.doc]

Table S5. Oligonucleotide list

| Primer name | Sequence |
| --- | --- |
| Rrp7-pED-F | 5’-CAGGGGCCCGaagctATGGGTATTGAAGACATTAGC-3’ |
| Rrp7-pED-R | 5’-gccgcAAGCTGAATTTTAAGTGTATGGATTGAATTTTC-3’ |
| Rrp7N162pEDF | 5’-ccatcaccatcatcaccacGGTATTGAAGACATTAGCG-3’ |
| Rrp7N162pEDR | 5’-CGCTAATGTCTTCAATACCgtggtgatgatggtgatgg-3’ |
| Rrp7C66F | 5’-GTTCCAGGGGCCCGaagctAACAAAAATCCATTATCCA-3’ |
| Rrp7C66R | 5’-TGGATAATGGATTTTTGTTagcttCGGGCCCCTGGAAC-3’ |
| Rrp7C42F | 5’-GTTCCAGGGGCCCGAAGCTGATTTCTATAGATTTCAGG-3’ |
| Rrp7C42R | 5’-CCTGAAATCTATAGAAATCAGCTTCGGGCCCCTGGAAC-3’ |
| Rrp7-pFB-F | 5’-CATCGGGCGCGGATCGCCACCATGGGTATTGAAGACAT -3’ |
| Rrp7-pFB-R | 5’-ACTTCTCGACAAGCTGGATCCTTAAGTGTATGGATTGAATTTTC-3’ |
| Rrp7-pFBHTPP-F | 5’-CAAGGTCCAGAAGCTATGGGTATTGAAGACATTAGCG-3’ |
| Rrp7-pFBHTPP-R | 5’-ACTTCTCGACAAGCTGGATCCTTAAGTGTATGGATTGAATTTTC-3’ |
| Rrp7-HTP-F | 5'-AAGAAAGAATCAAGGTAATGAAAGCTAAGAGAAAATTCAATCCATACACT CGTACGCTGCAGGTCGAC-3' |
| Rrp7-HTP-R | 5'-TTATAGATATATTGAGATATGTTGAATATGATGACGAGGATGGTGGTTTA ATCGATGAATTCGAGCTCG-3' |
| UTP22-HTP-F | 5’-ATGAGATTGCTGCATTCGGGAATGACATGGTTATAAATTTTGAGACAGAT CGTACGCTGCAGGTCGAC-3’ |
| UTP22-HTP-R | 5’-TTAATATTATACAGATACTTCTAAAAGTTATGATTTTGTTGTTTATTCTAATCGATGAATTCGAGCTCG-3’ |
| UTP22-pFB-F | 5’-CGGATCGCCACCATGGCTACAAGTGTTAAAAGAAAAGCATC-3’ |
| UTP22-pFB-R | 5’-TCGACAAGCTGGATCCTAATCTGTCTCAAAATTTATAACCATGTC-3’ |
| Utp22-pFBNHIS-F | 5’-TCGGGCGCGGATCCACCATGcatcatcatcatcatcatGCTACAAGTGTTAAAAGAAAA-3’ |
| Utp22-pFBNHIS-R | 5’-TTTTCTTTTAACACTTGTAGCatgatgatgatgatgatgCATGGTGGATCCGCGCCCGA-3’ |
| pGal-Rrp7-F | 5'-GAAGAGAACATTGAGAGGATGGTAAAGCAAGAGGCATTTAGAATTCGAGCTCGTTTAAAC-3' |
| pGal-Rrp7-R | 5'-TAAACCCGTTCTTCATGGCGCTAATGTCTTCAATACCCATTTTGAGATCCGGGTTTT-3' |
| pGal-snR30-F | 5'-TTTGAAACTCTCATCGTCGTGCATTTAACTTCTATGGTAAGAATTCGAGCTCGTTTAAAC-3' |
| pGal-snR30-R | 5-CCTTCCCTGTATAGTACCGAACTAGCACGAGACTATGGTTCATTTTGAGATCCGGGTTTT-3' |
| Rrp7pRS-F | 5’-CGGTATCGATAAGCTTATTATTTCTAACTTGGAAAC-3’ |
| Rrp7pRS-R | 5’-CGGGCTGCAGGAATTC TGTGCAGTTGTCATGCACATCA-3’ |
| Rrp7pRS-RBamHI | 5’-TAGAACTAGTGGATCCTGTGCAGTTGTCATGCACATCA-3’ |
| Rrp7F54A-F | 5’-CAACGAGTCTGACTGTTTGgcTTTGGTCAACCTTCCATTA-3’ |
| Rrp7F54A-R | 5’-TAATGGAAGGTTGACCAAAgcCAAACAGTCAGACTCGTTG-3’ |
| Rrp7Δ190-297-pRS415-F | 5’-ATTCATACACATATGGCATAAACCACCATCCTCGTC-3’ |
| Rrp7Δ190-297-pRS415-R | 5’-GACGAGGATGGTGGTTTATGCCATATGTGTATGAAT-3’ |
| Rrp7Δ1-89-pRS415-F | 5’-TCAGATAGAGTGTTACGGGAACGATGAATTTGGATTAC-3’ |
| Rrp7Δ1-89-pRS415-R | 5’-GTAATCCAAATTCATCGTTCCCGTAACACTCTATCTGA-3’ |
| Rrp7Δ1-156-pRS415-F | 5’-TCAGATAGAGTGTTACGGGGAATGGACATATACGACTC-3’ |
| Rrp7Δ1-156-pRS415-R | 5’-GAGTCGTATATGTCCATTCCCCGTAACACTCTATCTGA-3’ |
| Rrp7-F38D-F | 5’-GCTTCGTTGCATTTCATGgatGCTAAAAGACACCAGAGT-3’ |
| Rrp7-F38D-R | 5’-ACTCTGGTGTCTTTTAGCATCCATGAAATGCAACGAAGC-3’ |
| Rrp7-Δ95-105-F | 5’-ATATAACGATGAATTTGGAGATCTGATGTCCTCCACTG-3’ |
| Rrp7-Δ95-105-R | 5’-CAGTGGAGGACATCAGATCTCCAAATTCATCGTTATAT-3’ |
| Rrp7-Δ163-188-F | 5’-TGAATGGACATATACGACTGCAATTTTTGAACAGCGTG-3’ |
| Rrp7-Δ163-188-R | 5’-CACGCTGTTCAAAAATTGCAGTCGTATATGTCCATTCA-3’ |
| Utp22pRS-F | 5’-CGGTATCGATAAGCTTGCTTAGTGGTAGAGCGTTGCAC-3’ |
| Utp22pRS-R | 5’-CGGGCTGCAGGAATTC AAGGTCTTTGTATATTTTCTGC-3’ |
| Utp22pRS-RBamHI | 5’-TAGAACTAGTGGATCCAAGGTCTTTGTATATTTTCTGC-3’ |
| Utp22ΔD4-F | 5’-TGTGACAAATTAGTCACCGTTTGGTCTACATCCTCA-3’ |
| Utp22ΔD4-R | 5’-TGAGGATGTAGACCAAACGGTGACTAATTTGTCACA-3’ |
| Utp22R656ER657E-F | 5’-GGGAATTAAATCTTCCTTAgaAgaATTCAAGGATGGCTCCATC-3’ |
| Utp22R656ER657E-R | 5’-GATGGAGCCATCCTTGAATtcTtcTAAGGAAGATTTAATTCCC-3’ |
| Utp22R656E-F | 5’-GGGAATTAAATCTTCCTTAgaaAGATTCAAGGATGGCTCCATC-3’ |
| Utp22R656E-R | 5’-GATGGAGCCATCCTTGAATCTTTCTAAGGAAGATTTAATTCCC-3’ |
| Utp22R657E-F | 5’-GGGAATTAAATCTTCCTTAAGAgaaTTCAAGGATGGCTCCATC-3’ |
| Utp22R657E-R | 5’-GATGGAGCCATCCTTGAATTCTCTTAAGGAAGATTTAATTCCC-3’ |
| Utp22R217E-F | 5’-AGGAGTTGTTCGAAAAAgAAGATTTCTTAAATTTC-3’ |
| Utp22R217E-R | 5’-GAAATTTAAGAAATCTTcTTTTTCGAACAACTCCT-3’ |
| Utp22R223E-F | 5’-AAGATTTCTTAAATTTCgaGTGCTTACACAAGAGGA-3’ |
| Utp22R223E-R | 5’-TCCTCTTGTGTAAGCACtcGAAATTTAAGAAATCTT-3’ |
| Utp22R316E-F | 5’-CCCAATAGAAACTGTATTgaAATAGCACAAGAAAGCAA-3’ |
| Utp22R316E-R | 5’-TTGCTTTCTTGTGCTATTtcAATACAGTTTCTATTGGG-3’ |
| Utp22K104EL105D-F | 5’-ATTCAAGTCTAATATTTTCgAGgacCAAATTGACGAATTACTG-3’ |
| Utp22K104EL105D-R | 5’-CAGTAATTCGTCAATTTGGTCCTCGAAAATATTAGACTTGAAT-3’ |
| Utp22E109K-F | 5’-TTTCAAGTTACAAATTGACaAATTACTGGAGCAAGTGAA-3’ |
| Utp22E109K-R | 5’-TTCACTTGCTCCAGTAATTTGTCAATTTGTAACTTGAAA-3’ |
| Utp22K104EL105DE109K-F | 5’-TTCgAGgacCAAATTGACaAATTACTGGAGCAAGTGA-3’ |
| Utp22K104EL105DE109K-R | 5’-TCACTTGCTCCAGTAATTtGTCAATTTGgtcCTcGAA-3’ |
| Utp22D204A-F | 5’-CTAACGGCTCTTCCATTGcTACTTTACTAACAATGCCA-3’ |
| Utp22D204A-R | 5’-TGGCATTGTTAGTAAAGTAGCAATGGAAGAGCCGTTAG-3’ |
|  |  |
| FLAG-pRSNB-F | 5’-CGGGCTGCAGGAATTCGACTACAAGGACGACGATGACAAGtaaGATATCAAGCTTTGGAC-3’ |
| FLAG-pRSNB-R | 5’-GTCCAAAGCTTGATATCTTACTTGTCATCGTCGTCCTTGTAGTCGAATTCCTGCAGCCCG -3’ |
| Rrp7-pRSNB-F | 5’-TTAACGTCAAGGAGAAAAAACCCCGGATCCATGGGTATTGAAGACATTAG-3’ |
| Rrp7-pRSNB-R | 5’-CTTGTCATCGTCGTCCTTGTAGTCGAATTCAGTGTATGGATTGAATTTTC-3’ |
| Rrp7N189-pRSNB-F | 5’-TTAACGTCAAGGAGAAAAAACCCCGGATCCATTTTTGAACAGCGTGAAGC-3’ |
| Rrp7N189-pRSNB-R | 5’-CTTGTCATCGTCGTCCTTGTAGTCGAATTCTGCCATATGTGTATGAATAT-3’ |
| Rrp7Δ1-89-pRSNB-F | 5’-GAGAAAAAACCCCGGATCCAACGATGAATTTGGATTAC-3’ |
| Rrp7Δ1-89-pRSNB-R | 5’-GTAATCCAAATTCATCGTTGGATCCGGGGTTTTTTCTC-3’ |
| Rrp7Δ1-156-pRSNB-F | 5’-GAGAAAAAACCCCGGATCCGAATGGACATATACGACTC-3’ |
| Rrp7Δ1-156-pRSNB-R | 5’-GAGTCGTATATGTCCATTCGGATCCGGGGTTTTTTCTC-3’ |
|  |  |
| Utp22-pGBKT7-F | 5’-CTGCATATGGCCATGGCTACAAGTGTTAAAAGA-3’ |
| Utp22-pGBKT7-R | 5’-GCAGGTCGACGGATCCTAATCTGTCTCAAAATTTA-3’ |
| Rrp7-pGADT7-F | 5’-CAGATTACGCTCATATGGGTATTGAAGACATTAG-3’ |
| Rrp7-pGADT7-R | 5’-CGAGCTCGATGGATCTTAAGTGTATGGATTGAAT-3’ |
| Rrp7-190-pGADT7-F | 5’-CAGATTACGCTCATATGGCAATTTTTGAACAGCGT-3’ |
| Rrp7-189-pGADT7-R | 5’-CGAGCTCGATGGATCTTATGCCATATGTGTATGAAT-3’ |
| Rrp7Δ1-89-pGADT7-F | 5’-CGTACCAGATTACGCTCATAACGATGAATTTGGATTAC-3’ |
| Rrp7Δ1-89-pGADT7-R | 5’-GTAATCCAAATTCATCGTTATGAGCGTAATCTGGTACG-3’ |
| Rrp7Δ1-156-pGADT7-F | 5’-CGTACCAGATTACGCTCATGAATGGACATATACGACTC-3’ |
| Rrp7Δ1-156-pGADT7-R | 5’-GAGTCGTATATGTCCATTCATGAGCGTAATCTGGTACG-3’ |
