# Supplementary material for: An RNA-Binding Complex Involved in Ribosome Biogenesis Contains a Protein with Homology to tRNA CCA-Adding Enzyme
Source: PLoS Biol. 2013 Oct 1;11(10):e1001669. doi: 10.1371/journal.pbio.1001669 (PMC3794860; doi:10.1371/journal.pbio.1001669)
Supplement: Table S6 — Plasmid list. (DOC) [file pbio.1001669.s010.doc]

Table S6. Plasmid list

| Plasmids | Features | Information |
| --- | --- | --- |
| pFastBac1 |  | Invitrogen |
| pFastBac1-HTPP | An N-terminal His6-tag followed by a PreScission cleavage site | The DNA encoding a His6-tag and PreScission cleavage site was chemically synthesized and inserted into the pFastBac1 vector between BamHI and HindIII sites. |
| pFastBac1-Rrp7 | Rrp7 with no tag | The Rrp7 gene was PCR amplified from genomic DNA with primers Rrp7-pFB-F and Rrp7-pFB-R and inserted by In-fusion into pFastBac1 linearized with BamHI and HindIII. A Kozak sequence and an NcoI site were included in Rrp7-pFB-F. A BamHI site was included in Rrp7-pFB-R. |
| pFastBac1-HTPP-Rrp7 | Rrp7 with an N-terminal His6-tag and a PreScission cleavage site | The Rrp7 gene was PCR amplified from genomic DNA with primers Rrp7-pFBHTPP-F and Rrp7-pFBHTPP-R and inserted by In-fusion into pFastBac1-HTPP linearized with HindIII. |
| pFastBac1-Utp22 | Utp22 with no tag | The Utp22 gene was PCR amplified from genomic DNA with primers UTP22-pFB-F and UTP22-pFB-R and inserted by In-fusion into pFastBac1-Rrp7 linearized with NcoI and BamHI. |
| pFastBac1-His-Utp22 | Utp22 with an N-terminal noncleavable His_6_-tag | A His_6_-tag was inserted into pFastBacI-Utp22 by QuikChange with primers Utp22-pFBNHIS-F and Utp22-pFBNHIS-R. |
| pETDuet-SMT3 |  | Engineered from pETDuet-1 |
| pETDuet-SMT3-Rrp7 | Full-length Rrp7 with an N-terminal His6-SMT3 tag followed by a PreScission cleavage site | The Rrp7 gene was PCR amplified from genomic DNA with primers Rrp7-pED-F and Rrp7-pED-R and inserted by In-fusion into pETDuet-SMT3 linearized with HindIII. |
| pETDuet-Rrp7-1-162 | Rrp7 1-162 with an N-terminal noncleavable His6-tag. | pETDuet-SMT3-Rrp7 was mutagenized by QuikChange with primers Rrp7N162pEDF and Rrp7N162pEDR. |
| pETDuet-SMT3-Rrp7-189-297 | Rrp7 189-297 with an N-terminal His6-SMT3 tag followed by a PreScission cleavage site. | pETDuet-SMT3-Rrp7 was mutagenized by QuikChange with primers Rrp7C66F and Rrp7C66R. |
| pETDuet-SMT3-Rrp7-257-297 | Rrp7 257-297 with an N-terminal His6-SMT3 tag followed by a PreScission cleavage site. | pETDuet-SMT3-Rrp7 was mutagenized by QuikChange with primers Rrp7C42F and Rrp7C42R. |
|  |  |  |
| pRS416 | CEN; URA3 | Brachmann et al 1998 |
| pRS415 | CEN; LEU2 | Brachmann et al 1998 |
| pRS416-Rrp7 | CEN; URA3; Rrp7 ORF including 365 nt before start and 193 nt after stop codon | The Rrp7 gene was PCR amplified from genomic DNA with primers Rrp7pRS-F and Rrp7pRS-R and inserted by In-fusion into pRS416 linearized with HindIII and EcoRI. |
| pRS415-Rrp7 | CEN; LEU2; Rrp7 ORF including 365 nt before start codon and 193 nt after stop condon | The Rrp7 gene was PCR amplified from genomic DNA with primers Rrp7pRS-F and Rrp7pRS-RBamHI and inserted by In-fusion into pRS415 linearized with HindIII and BamHI. |
| pRS415-Rrp7F54A | pRS415-Rrp7 with Rrp7 mutation F54A | pRS415-Rrp7 was mutagenized by QuikChange with primers Rrp7F54A-F and Rrp7F54A-R. |
| pRS415-Rrp7Δ190-297 | pRS415-Rrp7 with deletion of Rrp7 residues 190-297 | pRS415-Rrp7 was mutagenized by QuikChange with primers Rrp7Δ190-297-pRS415-F and Rrp7Δ190-297-pRS415-R. |
| pRS415-Rrp7Δ1-89 | pRS415-Rrp7 with deletion of Rrp7 residues 1-89 | pRS415-Rrp7 was mutagenized by QuikChange with primers Rrp7Δ1-89-pRS415-F and Rrp7Δ1-89-pRS415-R. |
| pRS415-Rrp7Δ1-156 | pRS415-Rrp7 with deletion of Rrp7 residues 1-156 | pRS415-Rrp7 was mutagenized by QuikChange with primers Rrp7Δ1-156-pRS415-F and Rrp7Δ1-156-pRS415-R. |
| pRS415-Rrp7 F38D | pRS415-Rrp7 with Rrp7 mutation F54A | pRS415-Rrp7 was mutagenized by QuikChange with primers Rrp7-F38D-F and Rrp7-F38D-R. |
| pRS415-Rrp7Δ95-105 | pRS415-Rrp7 with deletion of Rrp7 residues 95-105 | pRS415-Rrp7 was mutagenized by QuikChange with primers Rrp7-Δ95-105-F and Rrp7-Δ95-105-R. |
| pRS415-Rrp7Δ163-188 | pRS415-Rrp7 with deletion of Rrp7 residues 163-188 | pRS415-Rrp7 was mutagenized by QuikChange with primers Rrp7-Δ163-188-F and Rrp7-Δ163-188-R. |
| pRS416-UTP22 | CEN; URA3;Utp22 ORF including 600nt before start codon and 269nt after stop codon | The Utp22 gene was PCR amplified from genomic DNA with primers Utp22pRS-F and Utp22pRS-R and inserted by In-fusion into pRS416 linearized with HindIII and EcoRI. |
| pRS415-UTP22 | CEN; LEU2; Utp22 ORF including 600nt before start codon and 269nt after stop codon | The Utp22 gene was PCR amplified from genomic DNA with primers Utp22pRS-F and Utp22pRS-RBamHI and inserted by In-fusion into pRS415 linearized with HindIII and BamHI. |
| pRS415-Utp22ΔD4 | pRS415-Utp22 with deletion of Utp22 residues 630-667 | pRS415-Utp22 was mutagenized by QuikChange with primers Utp22ΔD4-F and Utp22ΔD4-R. |
| pRS415-Utp22R656ER657E | pRS415-Utp22 with Utp22 mutations R656E and R657E | pRS415-Utp22 was mutagenized by QuikChange with primers Utp22R656ER657E-F and Utp22R656ER657E-R. |
| pRS415-Utp22R656E | pRS415-Utp22 with Utp22 mutation R656E | pRS415-Utp22 was mutagenized by QuikChange with primers Utp22R656E-F and Utp22R656E-R. |
| pRS415-Utp22R657E | pRS415-Utp22 with Utp22 mutation R657E | pRS415-Utp22 was mutagenized by QuikChange with primers Utp22R657E-F and Utp22R657E-R. |
| pRS415-Utp22K217E | pRS415-Utp22 with Utp22 mutation K217E | pRS415-Utp22 was mutagenized by QuikChange with primers Utp22R217E-F and Utp22R217E-R. |
| pRS415-Utp22R223E | pRS415-Utp22 with Utp22 mutation R223E | pRS415-Utp22 was mutagenized by QuikChange with primers Utp22R223E-F and Utp22R223E-R. |
| pRS415-Utp22R316E | pRS415-Utp22 with Utp22 mutation R316E | pRS415-Utp22 was mutagenized by QuikChange with primers Utp22R316E-F and Utp22R316E-R. |
| pRS415-Utp22 K217ER223E | pRS415-Utp22 with Utp22 mutation K217E and R223E | pRS415-Utp22K217E was mutagenized by QuikChange with primers Utp22R223E-F and Utp22R223E-R. |
| pRS415-Utp22 K217ER316E | pRS415-Utp22 with Utp22 mutation K217E and R316E | pRS415-Utp22K217E was mutagenized by QuikChange with primers Utp22R316E-F and Utp22R316E-R. |
| pRS415-Utp22 R223ER316E | pRS415-Utp22 with Utp22 mutation R223Eand R316E | pRS415-Utp22R223E was mutagenized by QuikChange with primers Utp22R316E-F and Utp22R316E-R. |
| pRS415-Utp22 K217ER223ER316E | pRS415-Utp22 with Utp22 mutations K217E, R223E and R316E | pRS415-Utp22 K217ER223E was mutagenized by QuikChange with primers Utp22R316E-F and Utp22R316E-R. |
| pRS415-Utp22 K104EL105D | pRS415-Utp22 with Utp22 mutation K104E and L105D | pRS415-Utp22 was mutagenized by QuikChange with primers Utp22K104EL105D-F and Utp22K104EL105D-R |
| pRS415-Utp22 E109K | pRS415-Utp22 with Utp22 mutation E109K | pRS415-Utp22 was mutagenized by QuikChange with primers Utp22E109K-F and Utp22E109K-R |
| pRS415-Utp22 K104EL105DE109K | pRS415-Utp22 with Utp22 mutations K104E, L105D and E109K | pRS415-Utp22 K104EL105D was mutagenized by QuikChange with primers Utp22K104EL105DE109K-F and Utp22K104EL105DE109K-R |
| pRS415-Utp22 UTP22D204A | pRS415-Utp22 with Utp22 mutation D204A | pRS415-Utp22 was mutagenized by QuikChange with primers Utp22D204A-F and Utp22D204A-R |
| pRSNB | 2µ, URA3, GAL1 promoter | The fragment containing the GAL1 promoter and yADH2 terminator was cleaved from plasmid pNB560 by PvuII and ligated into pRS426 linearized with PvuII. |
| pRSNB-FLAG | 2µ, URA3, GAL1 promoter, FLAG tag in MSC | The FLAG epitope was introducted by QuickChange into pRSNB with primers FLAG-pRSNB-F and FLAG-pRSNB-R. |
| pRSNB-Rrp7-FLAG | 2µ; URA3; RRP7 under GAL1 promoter | This construct was made by Transfer-PCR using pFastBac1-Rrp7 and pRSNB-FLAG as templates and primers Rrp7-pRSNB-F and Rrp7-pRSNB-R. |
| pRSNB-Rrp7N1-189-FLAG | pRSNB-Rrp7-FLAG with deletion of residues 190-256 | This construct was made by Transfer-PCR using pFastBac1-Rrp7 and pRSNB-FLAG as templates and primers Rrp7N189-pRSNB-F and Rrp7N189-pRSNB-R. |
| pRSNB-Rrp7Δ1-89 | pRSNB-Rrp7-FLAG with deletion of residues 1-89 | pRSNB-Rrp7-FLAG was mutagenized by QuikChange with primers Rrp7Δ1-89-pRSNB-F and Rrp7Δ1-89-pRSNB-R |
| pRSNB-Rrp7Δ1-156 | pRSNB-Rrp7-FLAG with deletion of residues 1-156 | pRSNB-Rrp7-FLAG was mutagenized by QuikChange with primers Rrp7Δ1-156-pRSNB-F and Rrp7Δ1-156-pRSNB-R |
| pGBKT7-Utp22 | 2µ; TRP1; GAL14(1-147) DNA-BD; Utp22 gene under the ADH1 promoter | The Utp22 gene was PCR amplified from plasmid pRS415-Utp22 with primers Utp22-pGBKT7-F and Utp22-pGBKT7-R and inserted by In-fusion into pGBKT7 linearized with NcoI and BamHI. |
| pGADT7-Rrp7 | 2µ; LEU2; GAL4(768-881)AD; Rrp7 gene under the ADH1 promoter | The Rrp7 gene was PCR amplified from plasmid pRS415-Rrp7 with primers Rrp7-pGADT7-F and Rrp7-pGADT7-F and inserted by In-fusion into pGADT7 linearized with NdeI and BamHI. |
| pGADT7-Rrp7 F38D | pGADT7-Rrp7 with mutation of F38D | pGADT7-Rrp7 was mutagenized by QuikChange with primers Rrp7-F38D-F and Rrp7-F38D-R |
| pGADT7-Rrp7Δ95-105 | pGADT7-Rrp7 with deletion of Rrp7 residues 95-105 | pGADT7-Rrp7 was mutagenized by QuikChange with primers Rrp7-Δ95-105-F and Rrp7-Δ95-105-R |
| pGADT7-Rrp7Δ163-188 | pGADT7-Rrp7 with deletion of Rrp7 residues 163-188 | pGADT7-Rrp7 was mutagenized by QuikChange with primers Rrp7-Δ163-188-F and Rrp7-Δ163-188-R |
| pGADT7-Rrp7Δ190-297 | pGADT7-Rrp7 with deletion of Rrp7 residues 190-297 | The Rrp7Δ190-297 gene was PCR amplified from plasmid pRS415-Rrp7 with primers Rrp7-pGADT7-F and Rrp7-189-pGADT7-R and inserted by In-fusion into pGADT7 linearized with NdeI and BamHI. |
| pGADT7-Rrp7Δ1-190 | pGADT7-Rrp7 with deletion of Rrp7 residues 1-190 | The Rrp7Δ1-190 gene was PCR amplified from plasmid pRS415-Rrp7 with primers Rrp7-190-pGADT7-F and Rrp7-pGADT7-R and inserted by In-fusion into pGADT7 linearized with NdeI and BamHI. |
| pGADT7-Rrp7Δ1-89 | pGADT7-Rrp7 with deletion of Rrp7 residues 1-89 | pGADT7-Rrp7 was mutagenized by QuikChange with primers Rrp7Δ1-89-pGADT7-F and Rrp7Δ1-89-pGADT7-R |
| pGADT7-Rrp7Δ1-156 | pGADT7-Rrp7 with deletion of Rrp7 residues 1-156 | pGADT7-Rrp7 was mutagenized by QuikChange with primers Rrp7Δ1-156-pGADT7-F and Rrp7Δ1-156-pGADT7-R |
