# Supplementary material for: An RNA-Binding Complex Involved in Ribosome Biogenesis Contains a Protein with Homology to tRNA CCA-Adding Enzyme
Source: PLoS Biol. 2013 Oct 1;11(10):e1001669. doi: 10.1371/journal.pbio.1001669 (PMC3794860; doi:10.1371/journal.pbio.1001669)
Supplement: Table S7 — Oligonucleotides for CRAC. (DOC) [file pbio.1001669.s011.doc]

Table S7. Oligonucleotides for CRAC

| Name | Sequence |
| --- | --- |
| 3’-linker  (miRNA Cloning Linker 1 from Integrated DNA Technologies, all DNA) | 5'-/5rApp/CTGTAGGCACCATCAAT/3ddC/-3' |
| 5’-linker  (Takara, all RNA) | 5'-/InvddT/GUUCAGAGUUCUACAGUCCGACGAUC-3' |
| DP3 | 5'-GATTGATGGTGCCTACAG-3' |
| DP5 | 5'-CAGAGTTCTACAGTCCGA-3' |
| SBS3 | 5'-CAAGCAGAAGACGGCATACGATTGATGGTGCCTACAG-3' |
| SBS5 | 5'-AATGATACGGCGACCACCGACAGGTTCAGAGTTCTACAGTCCGA-3' |
